# Transfer hydrogenation of aldehydes catalyzed by silyl hydrido iron complexes bearing a [PSiP] pincer ligand†

**DOI:** 10.1039/c8ra02606h

**Published:** 2018-04-17

**Authors:** Peng Zhang, Xiaoyan Li, Xinghao Qi, Hongjian Sun, Olaf Fuhr, Dieter Fenske

**Affiliations:** School of Chemistry and Chemical Engineering, Key Laboratory of Special Functional Aggregated Materials, Ministry of Education, Shandong University Shanda Nanlu 27 250100 Jinan People's Republic of China hjsun@sdu.edu.cn; Institut für Nanotechnologie (INT) und Karlsruher Nano-Micro-Facility (KNMF), Karlsruher Institut für Technologie (KIT) Hermann-von-Helmholtz-Platz 1, 76344 Eggenstein-Leopoldshafen Germany

## Abstract

The synthesis and characterization of a series of silyl hydrido iron complexes bearing a pincer-type [PSiP] ligand (2-R_2_PC_6_H_4_)_2_SiH_2_ (R = Ph (1) and ^i^Pr (5)) or (2-Ph_2_PC_6_H_4_)_2_SiMeH (2) were reported. Preligand 1 reacted with Fe(PMe_3_)_4_ to afford complex ((2-Ph_2_PC_6_H_4_)SiH)Fe(H)(PMe_3_)_2_ (3) in toluene, which was structurally characterized by X-ray diffraction. ((2-^i^Pr_2_PC_6_H_4_)SiH)Fe(H)(PMe_3_) (6) could be obtained from the reaction of preligand 5 with Fe(PMe_3_)_4_ in toluene. Furthermore, complex ((2-^i^Pr_2_PC_6_H_4_)Si(OMe))Fe(H)(PMe_3_) (7) was isolated by the reaction of complex 6 with 2 equiv. MeOH in THF. The molecular structure of complex 7 was also determined by single-crystal X-ray analysis. Complexes 3, 4, 6 and 7 showed good to excellent catalytic activity for transfer hydrogenation of aldehydes under mild conditions, using 2-propanol as both solvent and hydrogen donor. α,β-Unsaturated aldehydes could be selectively reduced to corresponding α,β-unsaturated alcohols. The catalytic activity of penta-coordinate complex 6 or 7 is stronger than that of hexa-coordinate complex 3 or 4.

## Abstract 2

The synthesis and characterization of a series of silyl hydrido iron complexes bearing a [PSiP] pincer ligand were reported. These complexes showed good to excellent catalytic activity for transfer hydrogenation of aldehydes under mild conditions.

## Graphical abstract

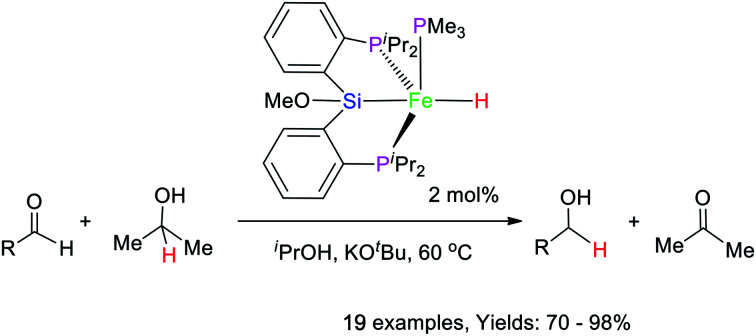

## 1 Introduction

Phosphine-based [PSiP] pincer complexes of transition metals have been studied extensively in recent years.^1–5^ In particular, they are involved as key intermediates in a variety of catalytic reactions of silicon compounds such as hydrosilylation, hydrocarboxylation of allenes and transfer hydrogenation.^6^ Because silyl ligands have stronger σ-donating characters and show a more potent trans-influence than commonly-used ligands in transition metal chemistry,^7^ the introduction of strong electron-donating and trans-labilizing silyl groups into tridentate ligand architectures may promote the formation of electron-rich and coordinatively-unsaturated complexes that exhibit novel reactivity with σ-bonds.^8^ Therefore, it is considered that silyl coordination compounds have potential applications in catalytic organic synthesis. In addition, changing phosphorus ligand would provide transition metal complexes with unique reactivity in catalytic reactions.^9,10^

Reduction of aldehydes and α,β-unsaturated aldehydes to alcohols is a fundamental and indispensable process for synthesis of a wide range of alcohols because a lot of alcohols are useful products and precursors for pharmaceutical, agrochemical, material and fine chemical industries.^11^ In most cases the transformation of aldehydes and α,β-unsaturated aldehydes to the related alcohols is a metal-catalyzed process. In this process, both H_2_ and alcohol can be used as reducing agents. In 2008, a series of new Pt(ii) pincer complexes bearing a pincer-type [PSiP] ligand (2-^i^Pr_2_PC_6_H_4_)_2_SiH_2_ were synthesized by Milstein's group. In addition, chloro-[PSiP]Pt complex was used to prepare silanol Pt(ii) pincer complex by hydrolytic oxidation.^12^ In 2013 Beller and co-workers reported the catalytic hydrogenation of aldehyde with H_2_. This catalytic system is chemoselective against ketone.^13^ However, that reaction required an elevated temperature (120 °C) and a high H_2_ pressure (30 bar). In the same year, three-coordinate iron(ii) and cobalt(ii) complexes bearing three new *N*-phosphinoamidinate ligands were synthesized by Turculet's group and the iron(ii) complexes as catalysts were used for hydrosilylation of carbonyl compounds with considerably low catalyst loading using 1 equiv. of PhSiH_3_.^14^ In 2014, Morris utilized three kinds of iron complexes bearing tetradentate PNNP ligands to realize successfully transfer hydrogenation of ketones and imines.^15^ In 2015, Hu described new iron pincer complexes. These complexes could activate H_2_ and catalyze selective transfer hydrogenation of aldehydes at room temperature under a low pressure of H_2_ (4 bar).^16^ Compared to the traditional hydrogenation reaction by the highly flammable molecular hydrogen employing precious metal (such as Au, Pt and Pd) catalysts,^17^ the reduction of aldehydes and α,β-unsaturated aldehydes *via* transfer hydrogenation using alcohol as both reaction solvent and source of hydrogen in the presence of cheap transition metal catalysts would be more promising because this is a safer, atom-efficient and environmentally-benign method. In most cases, 2-propanol as a conventional hydrogen donor solvent with a moderate boiling point (82 °C) serves as a reducing agent because it is stable and nontoxic. In addition, a strong base such as KO^*t*^Bu is usually necessary for most transfer hydrogenation processes in 2-propanol. In 2002 Crabtree developed a number of air-stable and moisture-insensitive Ir catalysts for efficient transfer hydrogenation.^18^ In 2006 Rashid and co-workers published several air-stable Ir complexes as effective catalysts for transfer hydrogenation of ketones under base-free conditions.^19^ In 2012 Colacino reported that four Ir(i) and Ir(iii) *N*-heterocyclic carbene (NHC) based complexes were used as catalysts in the reduction of aldehydes and ketones with glycerol.^20^

In this contribution, we have developed novel silyl hydrido iron [PSiP] pincer complexes for catalytic transfer hydrogenation of aldehydes and α,β-unsaturated aldehydes under mild conditions, using 2-propanol as both solvent and hydrogen donor. Furthermore, we compared the catalytic effects of these complexes with different phosphorus groups on the results of catalytic reactions.

## 2 Results and discussion

### 2.1 Reaction of Fe(PMe_3_)_4_ with (2-Ph_2_PC_6_H_4_)_2_SiRH (R = H (1) and Me (2))

In 2013, we reported the synthesis and characterization of a series of Ni, Co, and Fe complexes bearing a tridentate bis(phosphino)silyl ligand ((2-Ph_2_PC_6_H_4_)_2_SiMeH) (2) (eqn (1)). The silyl hydrido iron(ii) complex ((2-Ph_2_PC_6_H_4_)_2_SiMe)Fe(H)(PMe_3_)_2_ (4) was found to be an excellent catalyst for hydrosilylation of aldehydes and ketones under mild conditions.^21^1
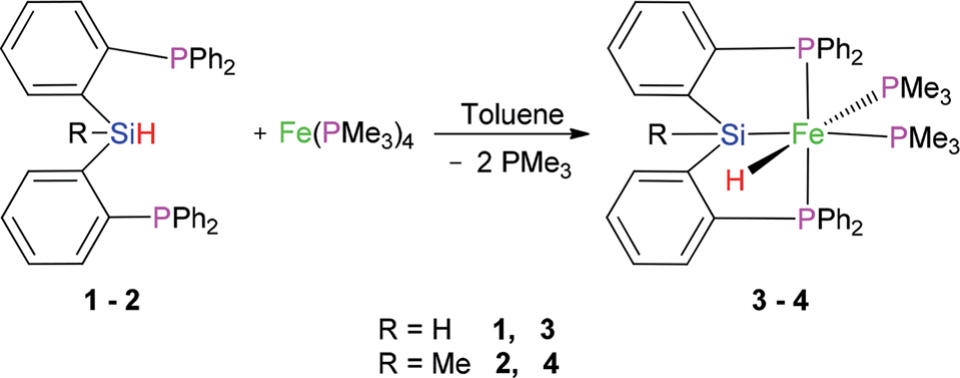


**Fig. 1:**
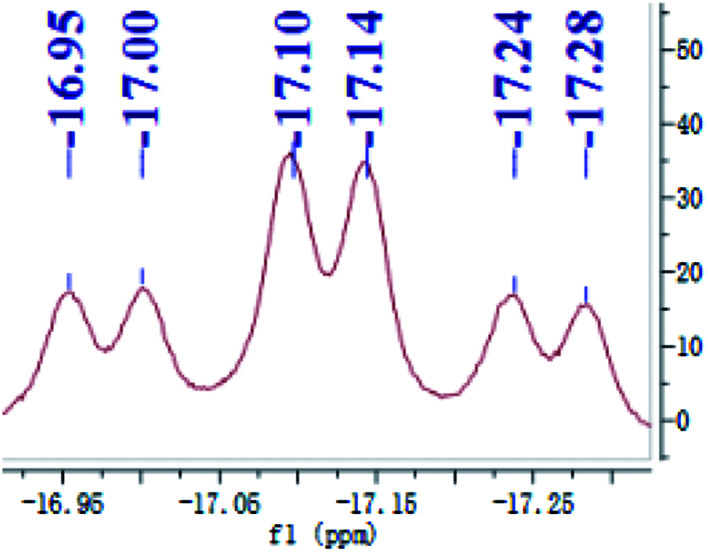
The hydrido resonance of complex 3 at −40 °C.

**Fig. 2:**
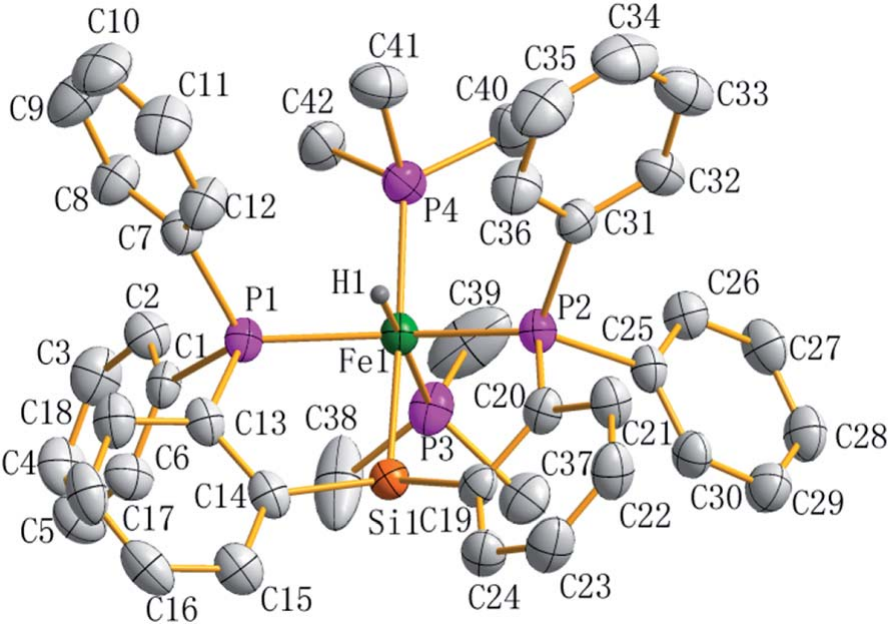
ORTEP plot of complex 3 at the 50% probability level (most of hydrogen atoms are omitted for clarity). Selected bond lengths (Å) and angles (deg), Fe1–P1 2.2050(1), Fe1–P2 2.1932(1), Fe1–P3 2.2513(1), Fe1–P4 2.2510(1), Fe1–Si1 2.2831(1), Fe1–H 1.5778(6), P3–Fe1–H1 172.56(5), P1–Fe1–P3 105.91(4), P2–Fe1–P1 146.42(4), P2–Fe1–P3 103.29(4), P1–Fe1–Si1 81.84(4), P2–Fe1–Si1 82.44(4), P3–Fe1–Si1 89.02(4), Si1–Fe1–H1 83.76(4), C14–Fe1–Si1 110.65(1), C19–Si1–Fe1 109.49(1).

Preligand 1 was treated with one equiv. of Fe(PMe_3_)_4_ in toluene at room temperature (eqn (1)). Complex 3 was isolated in a yield of 79% from diethyl ether at 0 °C. Orange bulk crystals of 3 suitable for X-ray diffraction were obtained from a concentrated THF solution layered with *n*-pentane at −20 °C. In the IR spectrum of 3, the typical *ν*(Fe–H) stretching band of complex 3 is found at 1836 cm^−1^ while the *ν*(Fe–H) stretching band of complex 4 is at 1870 cm^−1^.^19^ This bathochromic shift (34 cm^−1^) is caused through the replacement of the Me group in complex 4 by the H atom in complex 3 because the density of the electron cloud at the iron center in complex 3 is smaller than that in complex 4. The *ν*(Si–H) of complex 3 was recorded at 1992 cm^−1^ while the *ν*(Si–H) of preligand 1 was found at 2130 cm^−1^. In the ^1^H NMR spectrum of 3 at −40 °C, the characteristic hydrido signal was found at −17.12 ppm as a pseudo td peak with the coupling constants *J*_PH_ = 20 and 70 Hz (Fig. 1). The split pattern of the hydrido signal of 3 is same with that of 4.^19^ The proton signal of the Si–H bond of complex 3 appears at 5.72 ppm as d peak while the resonance of the Si–H bond in free preligand 1 was found at 5.87 ppm. Two signals at 0.97 and 0.45 ppm for two PMe_3_ ligands in the ^1^H NMR spectrum clearly indicate that the trimethylphosphine ligands are not chemically identical. It was found that two signals for PMe_3_ ligands and one signal for –P^i^Pr_2_ groups in the ^31^P NMR of complex 3 at −40 °C appeared at 5.2, 6.4 and 88.5 ppm in the integral ratio of 1 (PMe_3_) : 1 (PMe_3_) : 2 (–P^i^Pr_2_), respectively. The solid state structure of complex 3 shows a distorted hexa-coordinate octahedral geometry (Fig. 2). The axial angle P3–Fe1–H1 is 172.6°, slightly deviating from 180°. [Si1Fe1P1P4P2] are in the equatorial plane. In comparison with the structural data, the molecular structure of complex 3 is similar to that of complex 4.^19^ Fe1–H1 distance is 1.5776 Å. Owing to the strong trans-influence of H and Si atom, the distances Fe1–P3 (2.2513(1) Å) and Fe1–P4 (2.2510(1) Å) are significantly longer than the distances Fe1–P1 (2.2050(1) Å), Fe1–P2 (2.1932(1) Å).

### 2.2 Reaction of Fe(PMe_3_)_4_ with (2-^i^Pr_2_PC_6_H_4_)_2_SiH_2_ (5)



2

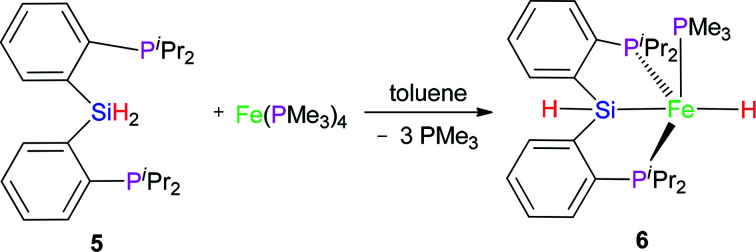




Complex 6 as pale yellow crystals was obtained from the reaction of 5 with Fe(PMe_3_)_4_ in toluene (eqn (2)). In the IR spectrum of complex 6, instead of the signal at 2140 cm^−1^ (*ν*(Si–H) for preligand 5), a new stretching band of the Si–H bond was found at 2051 cm^−1^. This large bathochromic shift (89 cm^−1^) indicates that the activation of the Si–H bond occurred. The *ν*(Fe–H) was registered at 1841 cm^−1^. In the ^1^H NMR spectrum of complex 6, the characteristic hydrido signal was found at −14.23 ppm as a td peak with the coupling constant *J*_PH_ = 18 and 72 Hz. The proton signal of the Si–H bond as multiplet appears at 5.91 ppm. Moreover, only one signal was identified at 1.11 ppm for one PMe_3_ ligand. In the ^31^P NMR of complex 6, two sets of signals were distinguished at 29.0 and 120.0 ppm, respectively, corresponding to the two kinds of P atoms in the integral ratio of 1 (PMe_3_) : 2 (–P^i^Pr_2_). Regrettably, no crystals of complex 6 suitable for X-ray diffraction were obtained. Compared with hexa-coordinate complex 3, the difference is that complex 6 is a penta-coordinated compound. Comparing isopropyl with phenyl group, the isopropyl group has a larger steric hindrance with stronger electron-donating ability than phenyl group. These two reasons make complex 6 penta-coordinated. Because complex 6 is a low-spin penta-coordination compound, which should have a tetragonal pyramid geometry. This can be further verified by the structure of complex 7.

### 2.3 Reaction of complex 6 with MeOH

The hydrido pincer iron(ii) complex 6 could react with MeOH to afford another hydrido pincer iron(ii) complex 7 (eqn (3)). In the IR spectrum of complex 7, the Fe–H vibration was found at 1845 cm^−1^, a little bit larger than that (1841 cm^−1^) of complex 6 because the MeO-group has electron-withdrawing ability. In the ^1^H NMR of complex 7 at −40 °C, the hydrido signal appeared at −11.10 ppm and split into a pseudo dddd peak due to the coupling effect of one PMe_3_ ligand and two chemically-identical –P^i^Pr_2_ groups. Moreover, the proton signal of the Si–H bond disappeared. There are two types of signals appearing at 22.2 and 106.2 ppm with a relative integral ratio of 1 (PMe_3_) : 2 (2× –P^i^Pr_2_) in the ^31^P NMR of complex 7. The related Pt chloride reacted with strong base to afford silyl ether Pt complex and the similar chemistry of Ru complex was also found by Stobart.^12^3
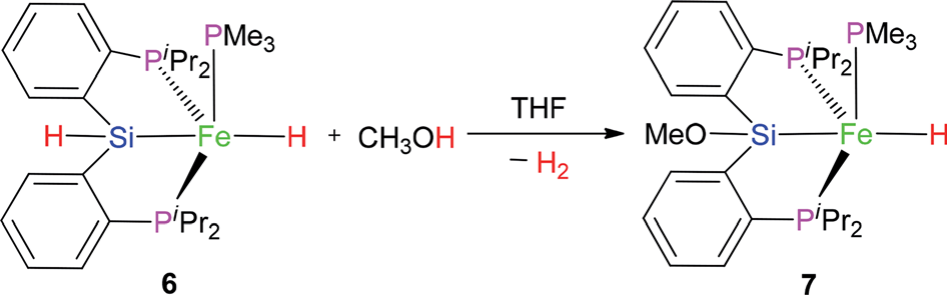


**Fig. 3:**
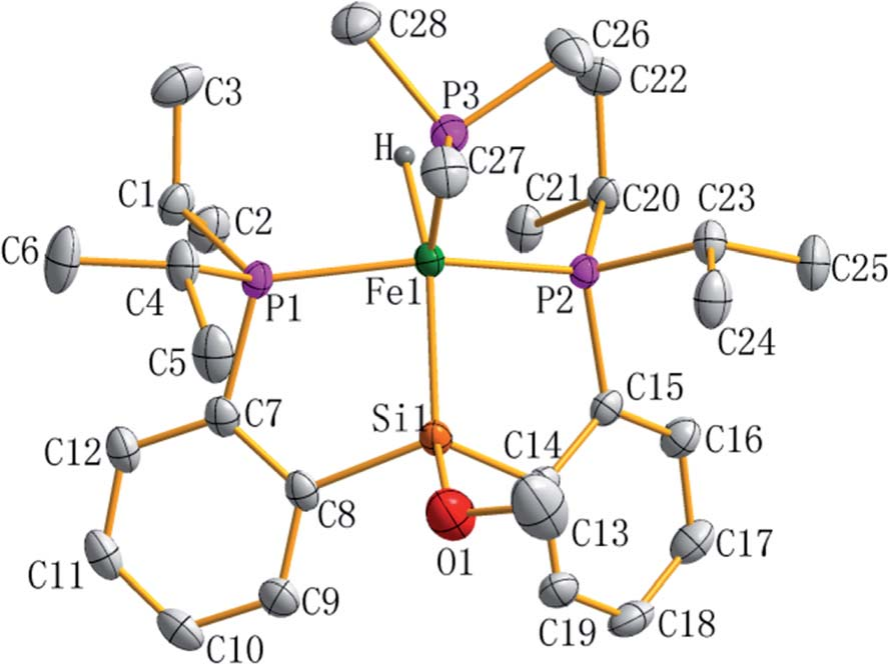
ORTEP plot of complex 7 at the 50% probability level (hydrogen atoms are omitted for clarity). Selected bond lengths (Å) and angles (deg): Fe1–Si1 2.2712(6), Fe1–P1 2.1998(6), Fe1–P2 2.1997(6), Fe1–P3 2.2183(7), Fe1–H 1.60(3), Si1–O1 1.672(2), O1–C13 1.410(4); P2–Fe1–P1 151.67(3), P1–Fe1–P3 101.43(2), P2–Fe1–P3 104.23(2), P1–Fe1–Si1 85.63(2), P2–Fe1–Si1 87.08(2), P3–Fe1–Si1 129.86(3), O1–Si1–Fe1 131.71(9), C13–O1–Si1 121.96(2), Si1–Fe1–H 151.5(1), Fe1–Si1–C14 105.94(7), C8–Fe1–Si1 107.05(7).

The molecular structure of complex 7 as a tetragonal pyramid (*τ*_5_ = 0.0105)^22^ with an iron atom in the center was confirmed by single crystal X-ray diffraction (Fig. 3). In this molecular structure, P3 is the apex point and [Fe1P1P2Si1H] is the base plane of this tetragonal pyramid. Fe1–H1 distance is 1.60(3) Å.

**Scheme 1:**
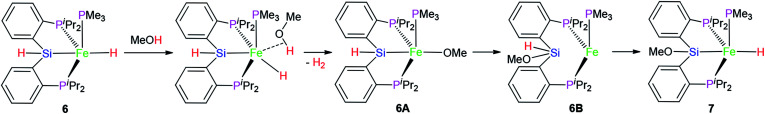


However, the similar reaction between complex 3 and MeOH did not occur. It is guessed that the difference in the reactivity between 3 and 6 might be caused by the vacant coordination in 6. This might allow for the coordination of MeOH (Scheme 1), followed by subsequent hydride protonation with the release of dihydrogen gas to form intermediate 6A (Scheme 1). The reductive elimination between Fe–Si and Fe–O bond affords intermediate 6B. Complex 7 was formed *via* oxidative addition of the Si–H bond at the iron(0) center of 6B. Complex 7 as complex 6 is also a penta-coordinate low-spin iron(ii) coordination compound.

### 2.4 Catalytic application of iron hydrides 3, 4 and 6, 7 in transfer hydrogenation of aldehydes



4

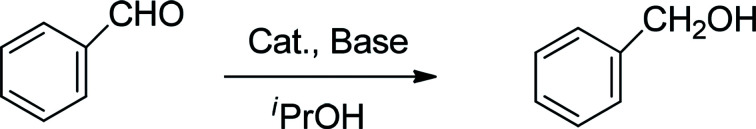




At the beginning, complex 7 as a catalyst was used to explore its catalytic application in the transfer hydrogenation of benzaldehyde (eqn (4)). The reaction was conducted with benzaldehyde as the test substrate using 2-propanol as the reaction solvent and source of hydrogen between 30–80 °C. When the reaction was performed without catalyst, no reduction product was obtained in the control experiment (entry 1, Table 1). If the catalyst loading was 1 mol%, the conversion declined (entry 4, Table 1). However, an excellent conversion (entry 3, Table 1) was observed in the presence of 2 mol% of complex 7. When the reaction temperature was 30 °C, the lower conversion was found (entry 11, Table 1). When the reaction temperature rose to 80 °C, the conversion declined sharply (entry 13, Table 1). And a grey precipitate appeared in the solution. It is guessed that the catalyst should have decomposed. Among NaO^*t*^Bu, Cs_2_CO_3_, K_2_CO_3_, Na_2_CO_3_, NaOH and KO^*t*^Bu, KO^*t*^Bu was the best base for this catalytic system (entries 3 and 6–10, Table 1). Without base, the reaction did not occur (entry 2, Table 1). At the given catalytic conditions, the reduction reaction was completely finished within 24 h. The conversion was lower when reaction time was shorter than 24 h (entry 12, Table 1). According to the experimental results in Table 1, the optimized catalytic reaction conditions can be summarized as follows: 60 °C, 24 hours and 2-propanol (5 mL), PhCHO (1.0 mmol) and 7 (0.02 mmol). The mole ratio the catalyst to base should be 1 : 1.

**Table 1:** Transfer hydrogenation of benzaldehyde with 7 as a catalyst^a^

Entry	Loading (mol%)	Base^b^	*T* (°C)	Time (h)	Conv.^c^ (%)
1	0	KO^*t*^Bu	60	24	0
2	2	None	60	24	0
3	2	KO^*t*^Bu	60	24	≥99
4	1	KO^*t*^Bu	60	24	81
5	5	KO^*t*^Bu	60	24	≥99
6	2	NaO^*t*^Bu	60	24	83
7	2	Cs_2_CO_3_	60	24	57
8	2	K_2_CO_3_	60	24	44
9	2	Na_2_CO_3_	60	24	21
10	2	NaOH	60	24	≤10
11	2	KO^*t*^Bu	30	24	61
12	2	KO^*t*^Bu	60	12	47
13	2	KO^*t*^Bu	80	24	19

a PhCHO (1.0 mmol), ^i^PrOH (5 mL).

b 7 : base = 1 : 1.

c Determined by GC with *n*-dodecane as internal standard.

Under the optimized reaction conditions, we expanded the scope of the aldehyde substrates bearing different functional groups (Table 2). As shown in Table 2, the reactions with 2 mol% of catalyst 7 at 60 °C in ^i^PrOH led to the corresponding alcohols with variable yields within 24 h. The substrates with the electron-withdrawing substituents, such as 2-fluorobenzaldehyde, 2-chlorobenzaldehyde, 2-bromobenzaldehyde, 4-fluorobenzaldehyde, 4-chlorobenzaldehyde and 4-bromobenzaldehyde could be reduced to the corresponding alcohols by using 2 mol% of catalyst in 24 hours (entries 3–8, Table 2). For the dihalogeno substrates, the aldehydes could be also converted to the corresponding products (entries 9 and 10, Table 2). When electron-donating group at *para*-position, moderate yield of the corresponding alcohol could be obtained from this catalytic system (entry 11, Table 2). With other aromatic aldehydes, moderate to good yields could be achieved (entries 13 and 14, Table 2). In addition, α,β-unsaturated aldehydes could be selectively reduced to the corresponding α,β-unsaturated alcohols in good yields (entries 15–19, Table 2)

**Table 2:** Transfer hydrogenation of aldehydes using 3, 4, 6 or 7 as catalyst^a^

Entry	Substrate	Catalyst	Isolated yield (%)
1	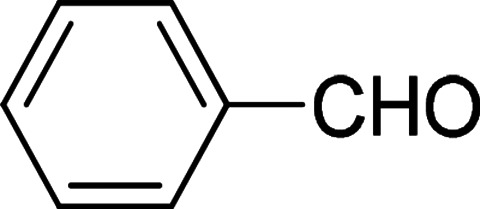	7	98
		6	94
		3	70
		4	73
2	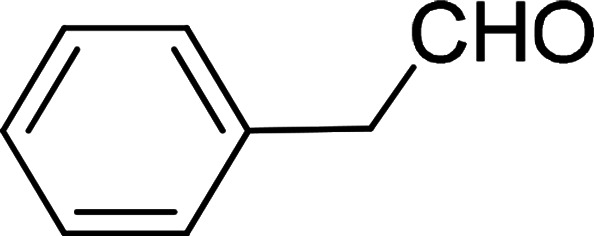	7	91
		6	83
		3	66
		4	74
3	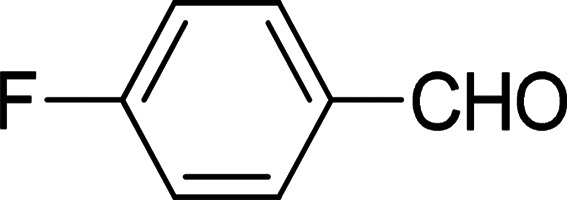	7	89
		6	85
		3	72
		4	70
4	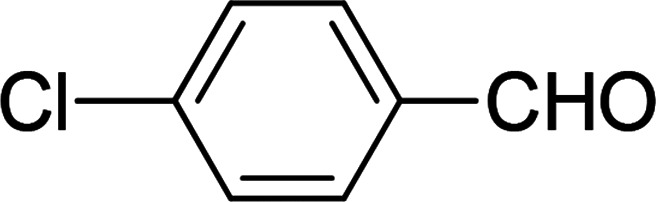	7	82
		6	83
		3	70
		4	71
5	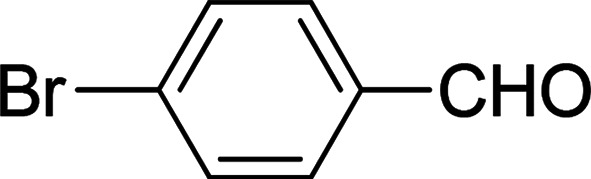	7	79
		6	82
		3	73
		4	70
6	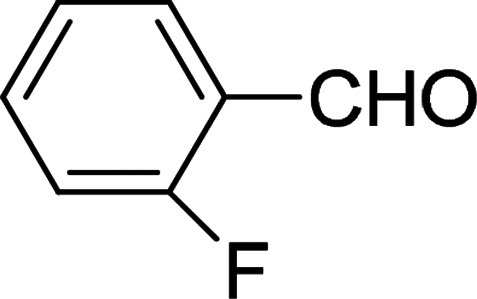	7	85
		6	84
		3	68
		4	72
7	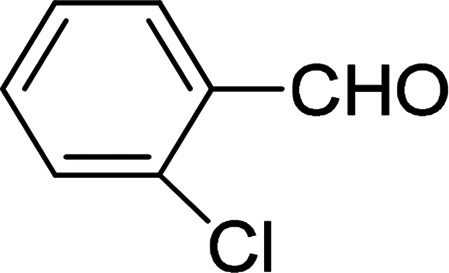	7	84
		6	80
		3	73
		4	71
8	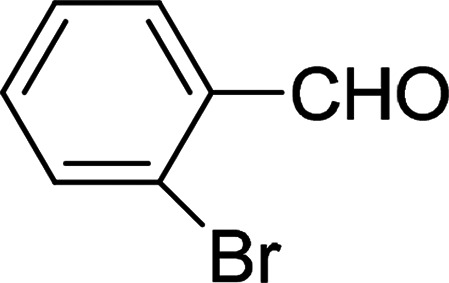	7	80
		6	81
		3	71
		4	66
9	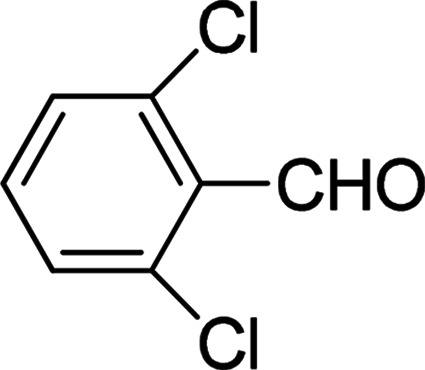	7	75
		6	77
		3	65
		4	61
10	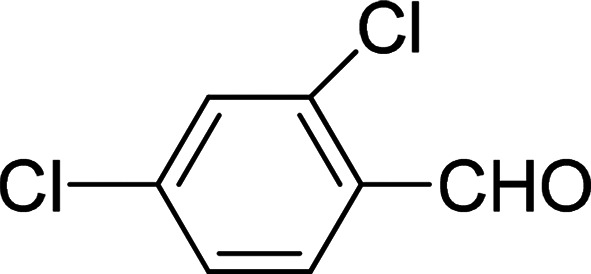	7	81
		6	83
		3	70
		4	66
11	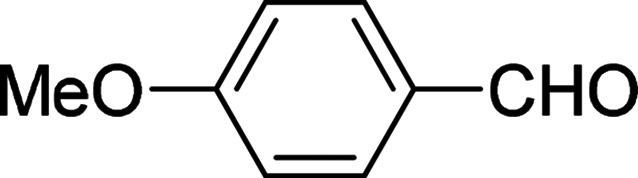	7	80
		6	81
		3	64
		4	61
12	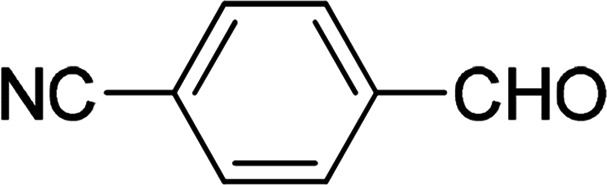	7	87
		6	84
		3	73
		4	76
13	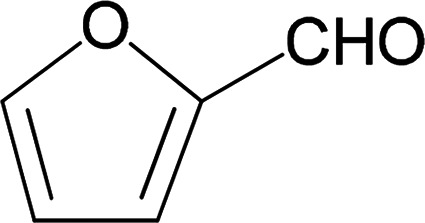	7	95
		6	91
		3	77
		4	71
14	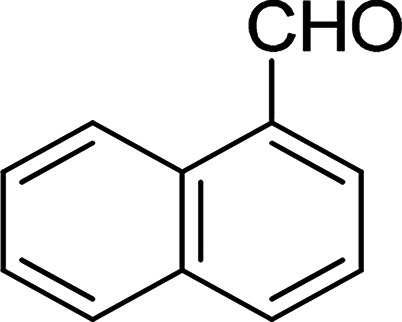	7	77
		6	75
		3	77
		4	75
15	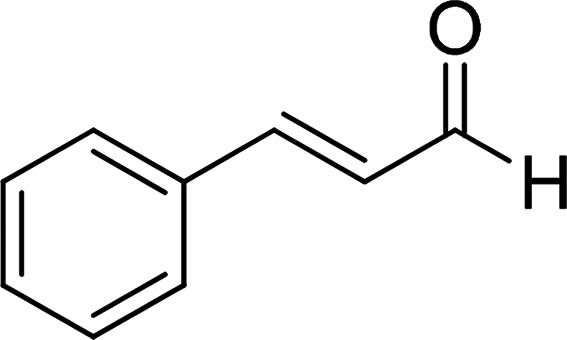	7	82
		6	79
		3	61
		4	67
16^b^	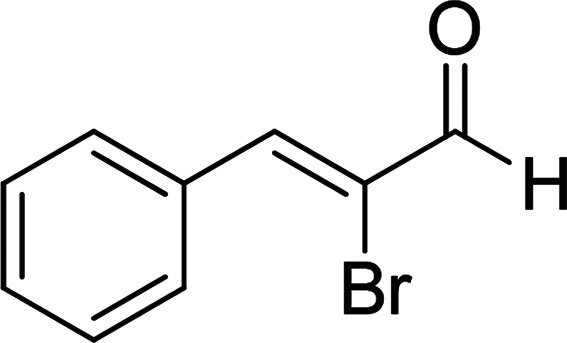	7	75
		6	71
		3	61
		4	66
17	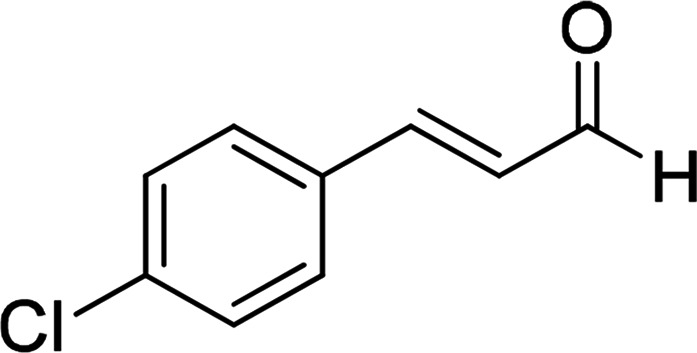	7	80
		6	76
		3	60
		4	69
18	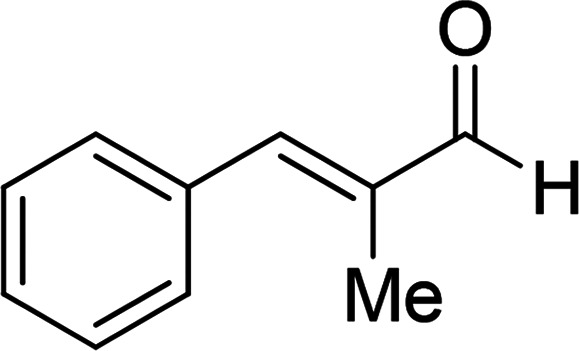	7	77
		6	74
		3	58
		4	63
19	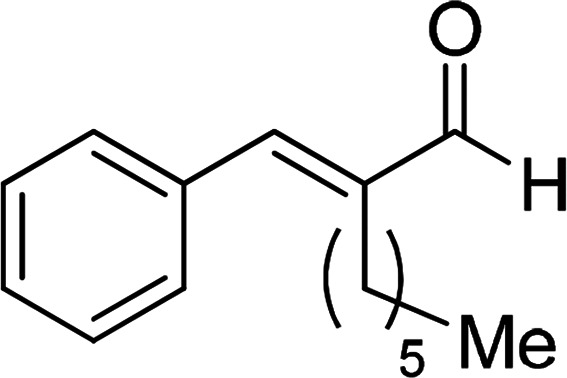	7	70
		6	72
		3	55
		4	61
20	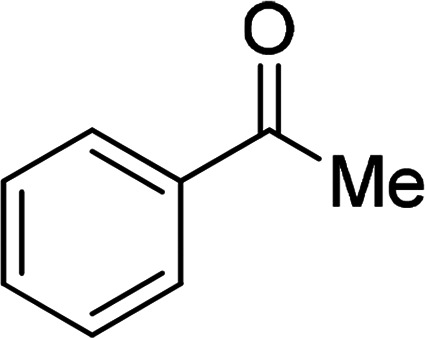	7	0
		6	0
		3	0
		4	0
21	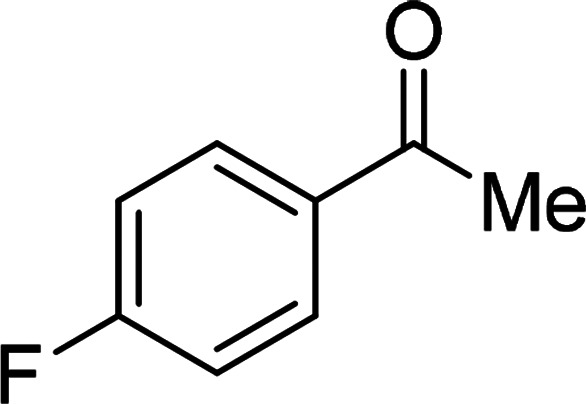	7	0
		6	0
		3	0
		4	0
22	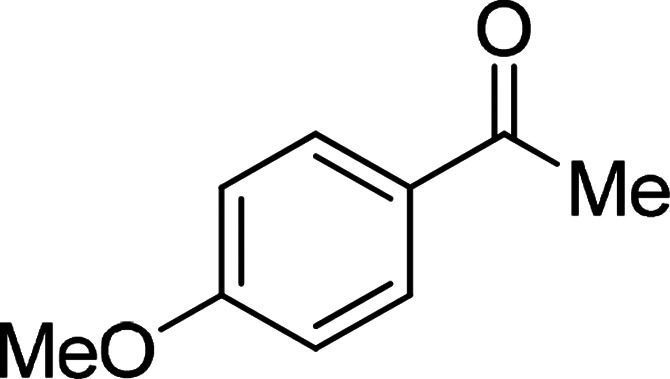	7	0
		6	0
		3	0
		4	0

a Substrate (1.0 mmol), KO^*t*^Bu (0.02 mmol), catalyst (0.02 mmol), ^i^PrOH (5 mL), 60 °C, 24 h.

b The reduced product is 3-phenylpro-2-yn-1-ol because the elimination occurred during the work-up.

Although complex 6 could also be used as catalyst for this catalytic system, the yields for the same substrates are lower than those of the reactions with complex 7 as catalyst in most cases. It is obvious that the introduction of MeO-group improves the catalytic activity of complex 7. From Table 2, we also know that the yields of the transformation with complex 3 or 4 as catalyst are significantly lower than those with complex 6 or 7 as catalyst. This is also caused by the different coordination number in complex 3 or 4 and 6 or 7. The hexa-coordinate complexes 3 and 4 are more stable than penta-coordinate complexes 6 and 7. As a final result, complex 6 or 7 has stronger catalytic activity than complex 3 or 4. Under these optimized catalytic conditions, the ketones could not be reduced to the corresponding alcohols with complex 3, 4, 6 or 7 as catalyst (entries 20–22, Table 2). It is considered that the steric effect plays a decisive role in this case.

**Scheme 2:**
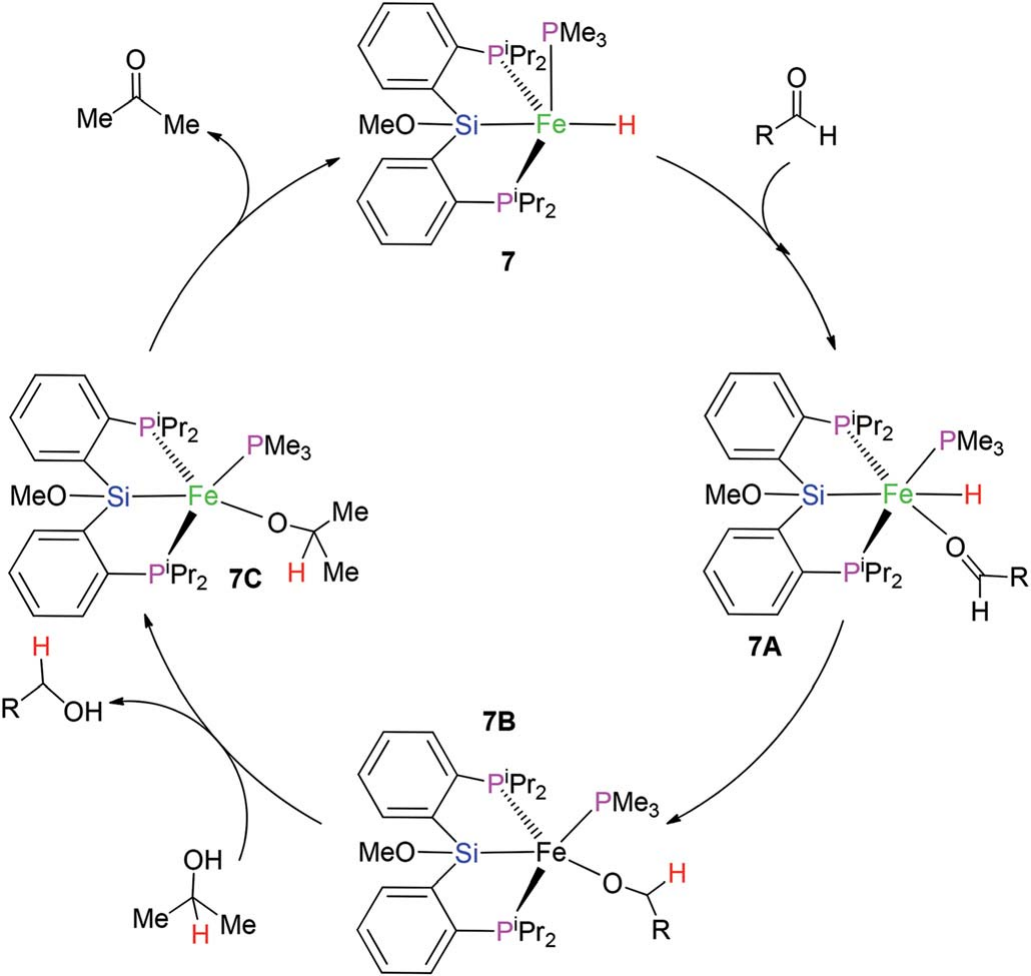
A plausible mechanism for transfer hydrogenation.

On the basis of the related report,^16^ a plausible mechanism for this catalytic system is proposed (Scheme 2). At first, complex 7 transforms to intermediate 7A*via* the coordination of carbonyl group in the aldehyde substrate. The nucleophilic attack of the hydrido hydrogen on the C atom of the carbonyl group gives rise to intermediate 7B. Again, the ligand substitution of RCH_2_O-group by Me_2_HC–O- group affords intermediate 7C with the formation of the final product RCH_2_OH. β-H elimination of the Me_2_HC–O-group provides acetone with the recovery of catalyst 7.

## 3 Conclusion

The silyl hydrido Fe(ii) complexs ((2-Ph_2_PC_6_H_4_)_2_HSi)Fe(H)(PMe_3_)_2_ (3) and ((2-^i^Pr_2_PC_6_H_4_)_2_HSi)Fe(H)(PMe_3_)_2_ (6) were synthesized by the oxidative addition of the Si–H bond of preligands (2-R_2_PC_6_H_4_)_2_SiH_2_ (R = Ph (1) and ^i^Pr (5)) to Fe(PMe_3_)_4_ respectively. Treatment of 6 with MeOH resulted in the formation of hydrido iron(ii) complex ((2-^i^Pr_2_PC_6_H_4_)_2_(MeO)Si)Fe(H)(PMe_3_) (7) *via* elimination of H_2_. Furthermore, we demonstrated transfer hydrogenation of aldehydes to alcohols using 3, 4, 6 and 7 as catalysts with ^i^PrOH as both solvent and hydrogen source in moderate to good yields. This catalytic system could be operated under mild conditions and has tolerance for some substrates with different substituents. α,β-Unsaturated aldehydes could be selectively reduced to corresponding α,β-unsaturated alcohols. The catalytic activity of penta-coordinate complex 6 or 7 is stronger than that of hexa-coordinate complex 3 or 4.

## 4 Experimental section

### 4.1 General procedures and materials

Standard vacuum techniques were used in the manipulation of volatiles and air-sensitive materials. Solvents were dried by metal sodium and distilled under nitrogen before use. The ligand 1 and 5 were prepared according to the literature.^9,10,12^ Fe(PMe_3_)_4_ was prepared according to literature procedures.^23^ Infrared spectra (4000–400 cm^−1^), as obtained from Nujol mulls between KBr disks, were recorded on a Bruker ALPHA FT-IR instrument. ^1^H, ^13^C{H}, ^31^P{H}, and ^29^Si{H} NMR spectra were recorded using Bruker Avance 300 MHz, 400 MHz, 500 MHz and 600 MHz spectrometers with C_6_D_6_ or THF-D_8_ as the solvent at the corresponding temperature. Melting points were measured in capillaries sealed under N_2_ and were uncorrected. Elemented analyses were carried out on an Elementar Vario EL III instrument.

### 4.2 Synthesis of 3

At −78 °C, Fe(PMe_3_)_4_ (0.31 g, 0.86 mmol) in 20 mL toluene was added to a solution of 1 (0.47 g, 0.86 mmol) in 40 mL of toluene. The mixture was warmed to room temperature and the color of solution has no obvious change. After stirred at room temperature for 24 h, the solution was evaporated to dryness at reduced pressure. The residue was washed by two portions of 10 mL of cold THF. Complex 3 (0.43 g, 0.47 mmol) was isolated as an orange powder in a yield of 79%. Crystals suitable for X-ray diffraction were obtained from *n*-pentane solution through recrystallization. dec.: > 147 °C. Anal. calc. for C_42_H_48_FeP_4_Si (760.62 g mol^−1^): C, 66.32; H, 6.36. Found: C, 66.67; H, 6.49. IR (Nujol mull, cm^−1^): 3048 (Ar–H), 1992 (Si–H), 1836 (Fe–H), 1583 (C=C), 940 (PMe_3_) cm^−1^. ^1^H NMR (500 MHz, THF-D_8_, 233 K, *δ*/ppm): −17.12 (td, ^2^*J*(PH) = 20.0, ^2^*J*(PH) = 70.0 Hz, 1H, Fe–H), 0.45 (s, PCH_3_, 9H), 0.97 (s, PCH_3_, 9H), 5.72 (d, ^2^*J*(PH) = 10.0 Hz, 1H, SiH); 6.58 (s, 2H, Ar–H), 7.07–7.37 (m, 20H, Ar–H), 7.63 (s, 4H, Ar–H), 8.39 (s, 2H, Ar–H). ^13^C NMR (75 MHz, C_6_D_6_, 298 K, *δ*/ppm): 19.3 (s, PCH_3_), 125.4 (s, Ar), 126.0 (t, ^3^*J*(PC) = 3.0 Hz, Ar), 127.0 (t, ^3^*J*(PC) = 1.5 Hz, Ar), 127.4 (s, Ar), 132.8 (t, ^3^*J*(PC) = 8.3 Hz, Ar), 133.2 (t, ^3^*J*(PC) = 9.0 Hz, Ar). ^31^P NMR (202.5 MHz, THF-D_8_, 233 K, *δ*/ppm): 5.2 (q, *J* = 30.4 Hz, PMe_3_, 1P), 6.4 (m, PMe_3_, 1P), 88.5 (t, *J* = 20.3 Hz, PPh_2_, 2P). ^29^Si NMR (79.45 MHz, C_6_D_6_, 298 K, *δ*/ppm): 68.8 (s).

### 4.3 Synthesis of 6

To a brown yellow solution of Fe(PMe_3_)_4_ (0.40 g, 1.11 mmol) in 20 mL of toluene was added a solution of 5 (0.42 g, 1.01 mmol) in 30 mL of toluene. The mixture was stirred at room temperature for 32 h. During this period, the reaction solution turned yellow. The volatiles were removed by vacuum. The viscous residue was extracted with *n*-pentane and diethyl ether. The pale yellow crystals of 6 were obtained from diethyl ether at 0 °C. Yield: 480 mg (65%). Crystals suitable for X-ray diffraction were obtained in pentane solution. dec.: > 127 °C. Anal. calc. for C_27_H_47_FeP_3_Si (548.51 g mol^−1^): C, 59.12; H, 8.64. Found: C, 58.87; H, 8.51. IR (Nujol mull, cm^−1^): 3058 (Ar–H), 2051 (Si–H), 1841 (Fe–H), 1554 (C=C), 950 (PMe_3_). ^1^H NMR (300 MHz, C_6_D_6_, 298 K, *δ*/ppm): −14.23 (td, ^2^*J*(PH) = 18.0 Hz, ^2^*J*(PH) = 72.0 Hz, 1H, Fe–H), 0.71 (q, ^2^*J*(PH) = 6.0 Hz, PCHCH_3_, 6H), 0.84–0.89 (m, PCHCH_3_, 12H), 0.92 (q, ^2^*J*(PH) = 6.0 Hz, PCHCH_3_, 6H), 1.11 (d, *J* = 3.0 Hz, PCH_3_, 9H), 1.75–1.89 (m, PCHCH_3_, 2H), 2.28–2.40 (m, PCHCH_3_, 2H), 5.86–5.95 (m, 1H, SiH); 6.95–6.99 (m, 2H, Ar–H), 7.04–7.09 (m, 2H, Ar–H), 7.14–7.16 (m, 2H, Ar–H), 8.03 (d, ^2^*J*(PH) = 6.0 Hz, 2H, Ar–H). ^13^C NMR (75 MHz, C_6_D_6_, 298 K, *δ*/ppm): 23.3 (dd, ^3^*J*(PC) = 7.5 Hz, ^3^*J*(PC) = 16.5 Hz, PCHCH_3_), 25.6 (s, PCHCH_3_), 26.6 (q, ^3^*J*(PC) = 6.0 Hz, PCH_3_), 126.4 (s, Ar), 131.4 (s, Ar), 132.5 (t, ^3^*J*(PC) = 9.0 Hz, Ar), 133.3 (t, ^3^*J*(PC) = 4.5 Hz, Ar), 133.9 (t, ^3^*J*(PC) = 4.5 Hz, Ar), 143.5 (s, Ar), 155.0 (t, ^3^*J*(PC) = 26.3 Hz, Ar). ^31^P NMR (121 MHz, C_6_D_6_, 298 K, *δ*/ppm): 29.0 (t, *J* = 27.8 Hz, PMe_3_, 1P), 120.0 (d, *J* = 27.8 Hz, P^i^Pr_2_, 2P). ^29^Si NMR (79.45 MHz, C_6_D_6_, 298 K, *δ*/ppm): 57.7 (s).

### 4.4 Synthesis of 7

At 0 °C, MeOH (0.044 g, 1.31 mmol) in 20 mL of THF was combined with 6 (0.38 g, 0.69 mmol) in 30 mL of THF. The solution was taken to room temperature and stirred for 24 h. The volatiles were removed at reduced pressure. The residue was extracted with *n*-pentane and diethyl ether. Complex 7 (247 mg) was isolated as pale yellow crystals in a yield of 62%. Crystals suitable for X-ray diffraction were obtained from the *n*-pentane solution. dec.:> 123 °C. Anal. calc. for C_28_H_49_FeOP_3_Si (578.52 g mol^−1^): C, 58.13; H, 8.54. Found: C, 58.40; H, 8.71. IR (Nujol mull, cm^−1^): 3053 (Ar–H), 1845 (Fe–H), 1583 (C=C), 943 (PMe_3_). ^1^H NMR (500 MHz, THF-D_8_, 233 K, *δ*/ppm): −11.10 (dddd, ^2^*J*(PH) = 90.0 Hz, 72.0 Hz, 45.0 Hz, 1H, Fe–H), 0.76 (q, ^2^*J*(PH) = 5.0 Hz, PCHCH_3_, 6H), 0.84 (q, ^2^*J*(PH) = 5.0 Hz, PCHCH_3_, 6H), 1.08 (q, ^2^*J*(PH) = 5.0 Hz, PCHCH_3_, 6H), 1.23 (q, ^2^*J*(PH) = 5.0 Hz, PCHCH_3_, 6H), 1.43 (d, ^2^*J*(PH) = 5.0 Hz, PCH_3_, 9H), 1.99 - 2.05 (m, PCHCH_3_, 2H), 2.72 (t, ^2^*J*(PH) = 5.0 Hz, PCHCH_3_, 2H), 3.39 (s, –OCH_3_, 3H), 7.17 (t, ^2^*J*(PH) = 5.0 Hz, 2H, Ar–H), 7.25 (t, ^2^*J*(PH) = 5.0 Hz, 2H, Ar–H),7.39 (d, ^2^*J*(PH) = 10.0 Hz, 2H, Ar–H), 7.83 (d, ^2^*J*(PH) = 5.0 Hz, 2H, Ar–H). ^31^P NMR (202.5 MHz, THF-D_8_, 298 K, *δ*/ppm): 22.2 (t, *J* = 24.3 Hz, PMe_3_, 1P), 106.2 (dd, *J* = 8.1 Hz, *J* = 24.3 Hz, P^i^Pr_2_, 2P). ^13^C NMR (100 MHz, C_6_D_6_, 298 K, *δ*/ppm): 17.1 (s, PCH_3_), 19.9 (s, PCHCH_3_), 30.0 (s, PCHCH_3_), 67.5 (s, OCH_3_), 126.48 (s, Ar), 128.83 (s, Ar), 131.2 (t, ^3^*J*(PC) = 8.0 Hz, Ar), 143.1 (t, ^3^*J*(PC) = 27.0 Hz, Ar), 157.81 (t, ^3^*J*(PC) = 23.0 Hz, Ar). ^29^Si NMR (79.45 MHz, C_6_D_6_, 298 K, *δ*/ppm): 42.2 (s).

### 4.5 General procedure for transfer hydrogenation of aldehydes

In 25 mL Schlenk tube containing a solution of 7 (0.02 mmol) in 5 mL of ^i^PrOH were added an aldehyde (1.0 mmol) and KO^*t*^Bu (0.02 mmol). The reaction mixture was stirred at 60 °C. The organic product was extracted with Et_2_O and further purified by chromatography.

### 4.6 X-Ray structure determinations

Crystallographic data for complexes 3 and 7 are summarized in the ESI.† Intensity data were collected on a Stoe Stadi Vari Cu diffractometer. Using Olex2,^24^ the structure was solved with ShelXS^25^ structure solution program using direct methods and refined with the ShelXL^26^ refinement package using least squares minimization. CCDC-1515026 (3) and 1490870 (7) contain supplementary crystallographic data for this paper.

## Conflicts of interest

There are no conflicts to declare.

## Supplementary Material

RA-008-C8RA02606H-s001

RA-008-C8RA02606H-s002
