# Supplementary material for: Transfer hydrogenation of aldehydes catalyzed by silyl hydrido iron complexes bearing a [PSiP] pincer ligand
Source: RSC Adv. 2018 Apr 17;8(25):14092–9. doi: 10.1039/c8ra02606h (PMC9079873; doi:10.1039/c8ra02606h)

Electronic Supplementary Material (ESI) for RSC Advances.
This journal is © The Royal Society of Chemistry 2018

Transfer Hydrogenation of Aldehydes Catalyzed by Silyl Hydrido Iron Complexes Bearing a [PSiP] Pincer Ligand

Peng Zhang,[a] Xiaoyan Li,[a] Xianghao Qi,[a] Hongjian Sun,*[,a] Olaf Fuhr,[b] Dieter Fenske[b]

[a] *School of Chemistry and Chemical Engineering, Key Laboratory of Special Functional Aggregated Materials, Ministry of Education, Shandong University, Shanda Nanlu 27, 250100 Jinan, People's Republic of China*

[b] *Institut für Nanotechnologie (INT) und Karlsruher Nano-Micro-Facility (KNMF), Karlsruher Institut für Technologie (KIT), Hermann-von-Helmholtz-Platz 1, 76344 Eggenstein-Leopoldshafen, Germany*

Table of contents

1 Table S1 Crystallographic Data for complexes **3** and **7**……..S2

2 IR, ^{1}H NMR, ^{31}P NMR, ^{13}C NMR and ^{29}Si spectra of complexes **3, 6** and **7**……..S3

3 NMR spectra of the catalytic products……..S10

1 Table S1 Crystallographic Data for complexes 3 and **7**

Complex	**3**	**7**
Formula	$C_{42}H_{48}FeP_4Si$	$C_{28}H_{49}FeOP_3Si$
Mz	760.62	578.52
crystal system	monoclinic	monoclinic
space group	$P2_1/c$	$P2_1/n$
a [Å]	11.7181(7)	13.5670(5)
b [Å]	18.9232(6)	16.7800(5)
c [Å]	35.1134(2)	13.9927(5)
α[°]	90	90
β[°]	91.084(4)	106.554(3)
γ[°]	90	90
V [$Å^3$]	7784.8(6)	3053.46(2)
T [K]	200.15	153.15
Z	8	4
μ[mm^{-1}]	5.169	0.709
total reflns	26696	15439
unique reflns	11341	5786
R_{int}	0.0446	0.0385
R1[I>2σ(I)]	0.0515	0.0352
wR(F^2)[I>2σ(I]	0.1312	0.0877
R1(all data)	0.0710	0.0498
wR(F^2) (all data)	0.1419	0.0924
GOF on F^2	1.029	0.973

2 IR, ^{1}H NMR, ^{31}P NMR, ^{13}C NMR and ^{29}Si NMR spectrum of complexes 3, 6 and 7

IR spectrum of complex **3**

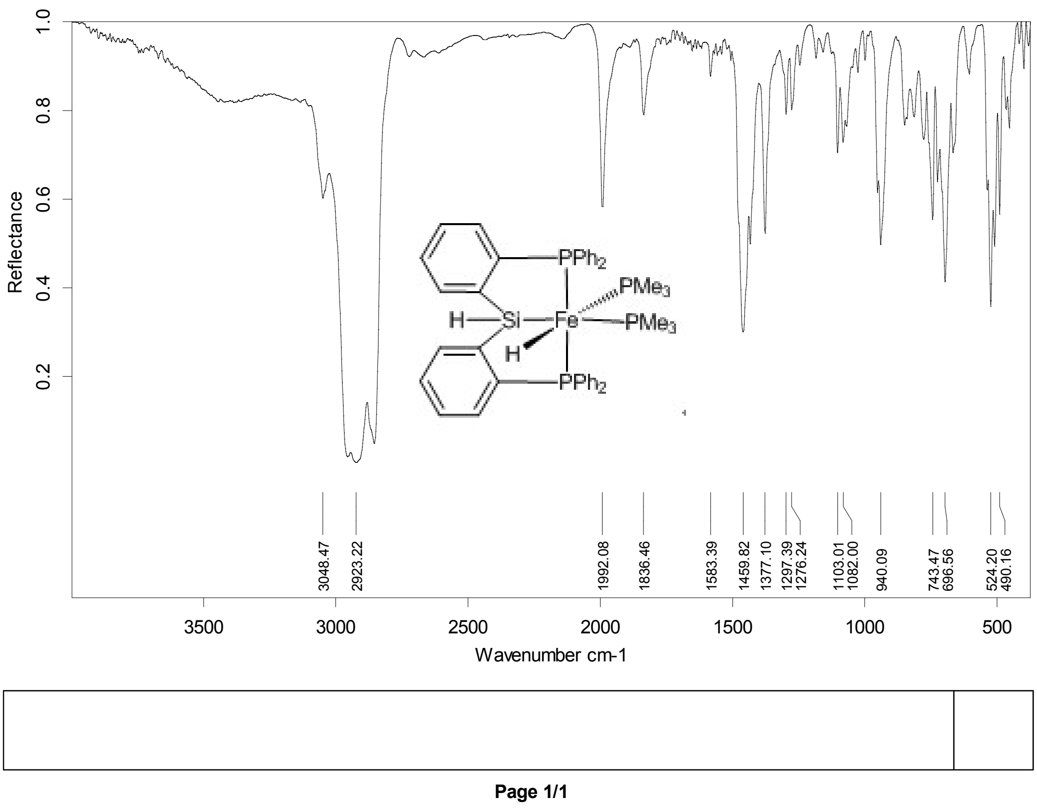

Fig. S1 IR spectrum of complex **3.**

^{1}H NMR of complex **3**

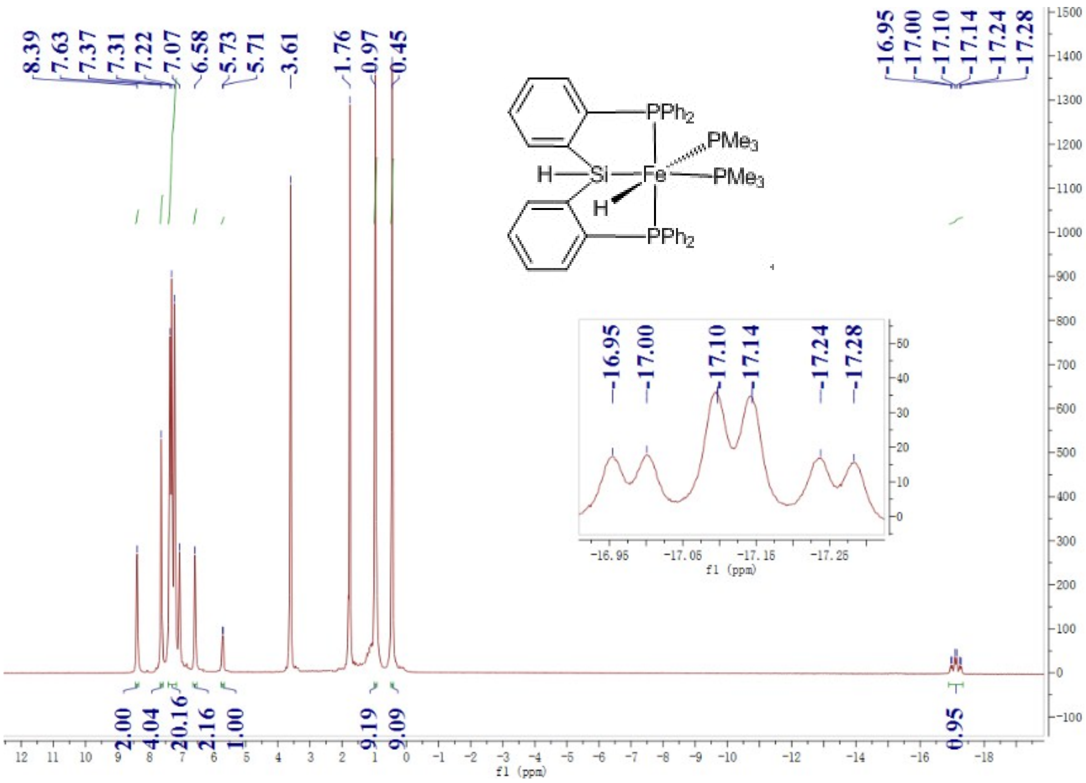

Fig. S2 ^{1}H NMR of complex **3** at -40℃

^{31}P NMR of complex **3**

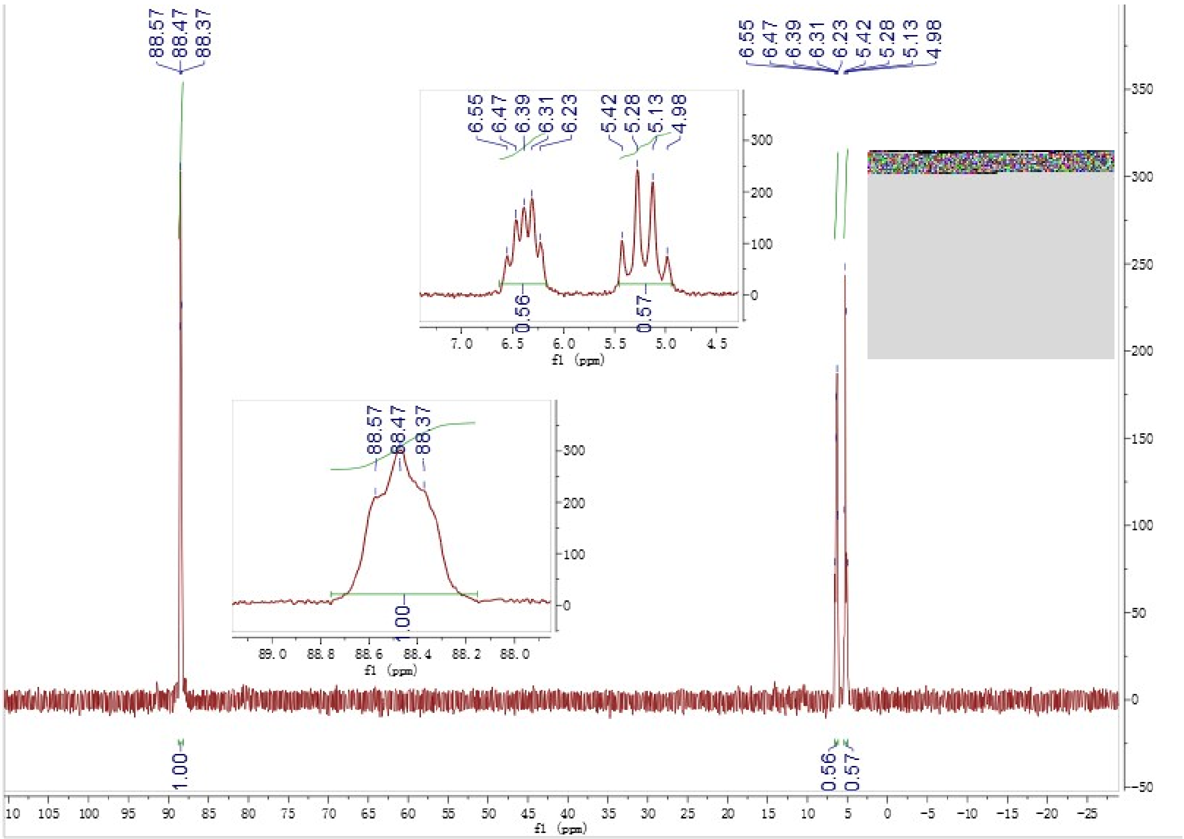

Fig. S3 ^{31}P NMR of complex **3** at -40℃

^{13}C NMR of complex **3**

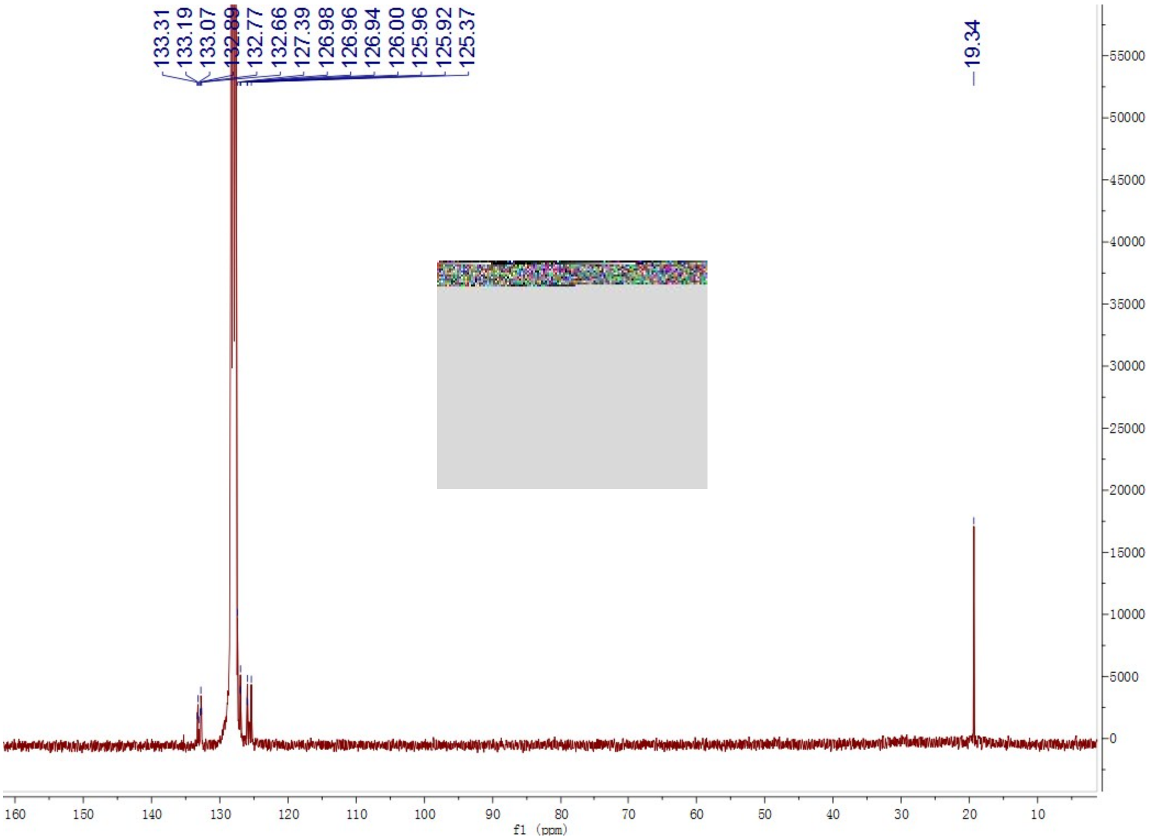

Fig. S4 ^{13}C NMR of complex **3.**

^{29}Si NMR of complex **3**

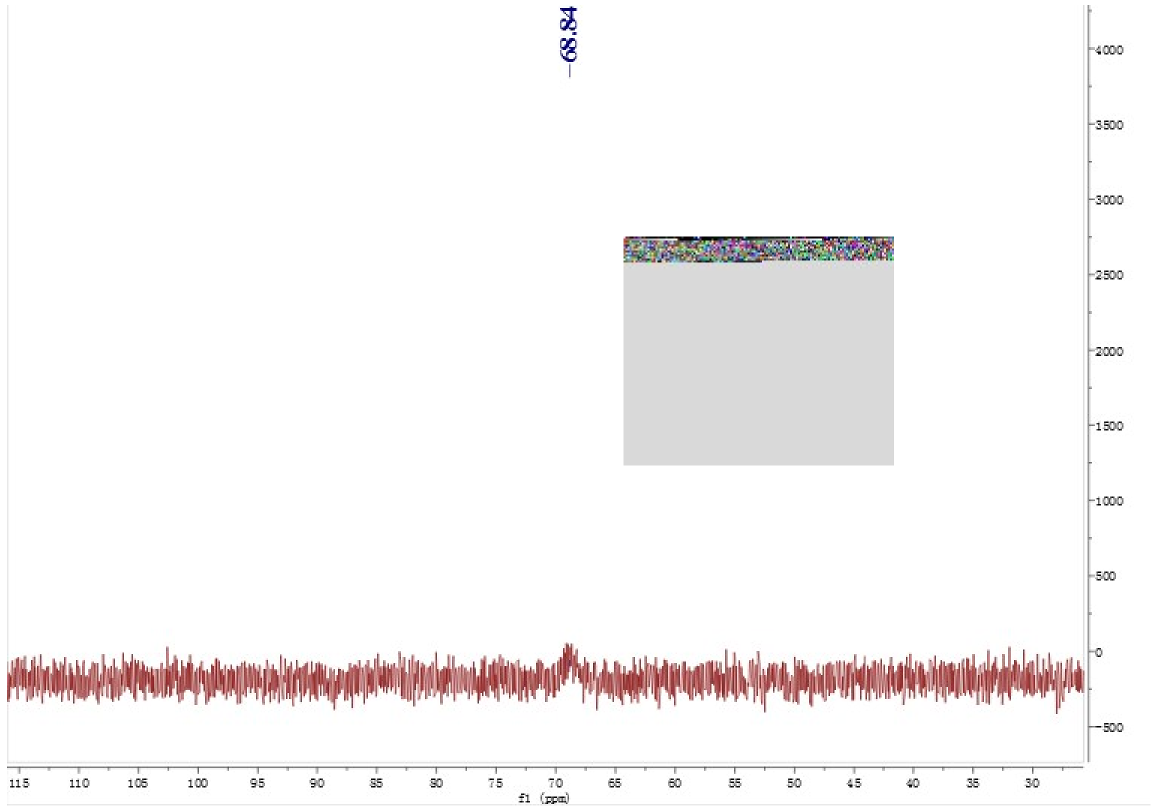

Fig. S5 ^{29}Si NMR of complex **3.**

IR spectrum of complex **6**

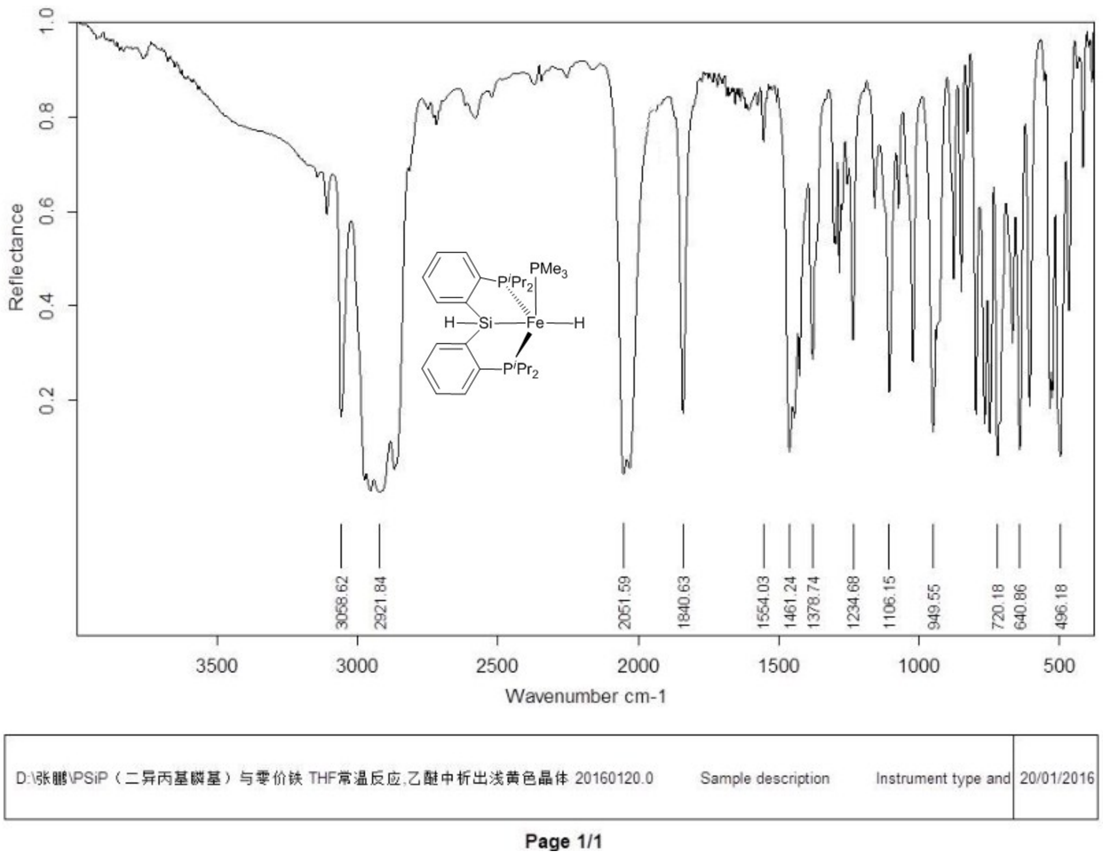

Fig. S6 IR spectrum of complex **6.**

^{1}H NMR of complex **6**

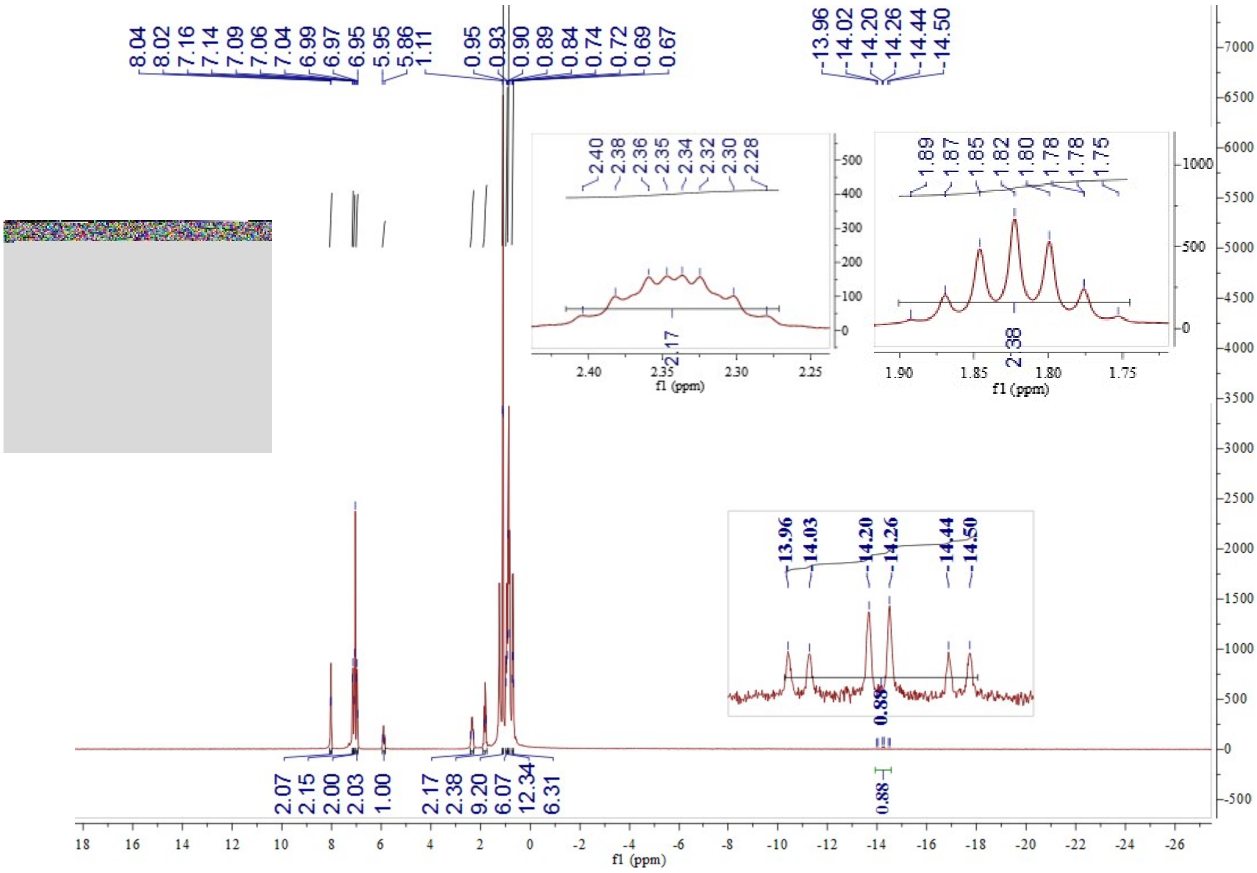

Fig. S7 ^{1}H NMR of complex **6.**

^{31}P NMR of complex **6**

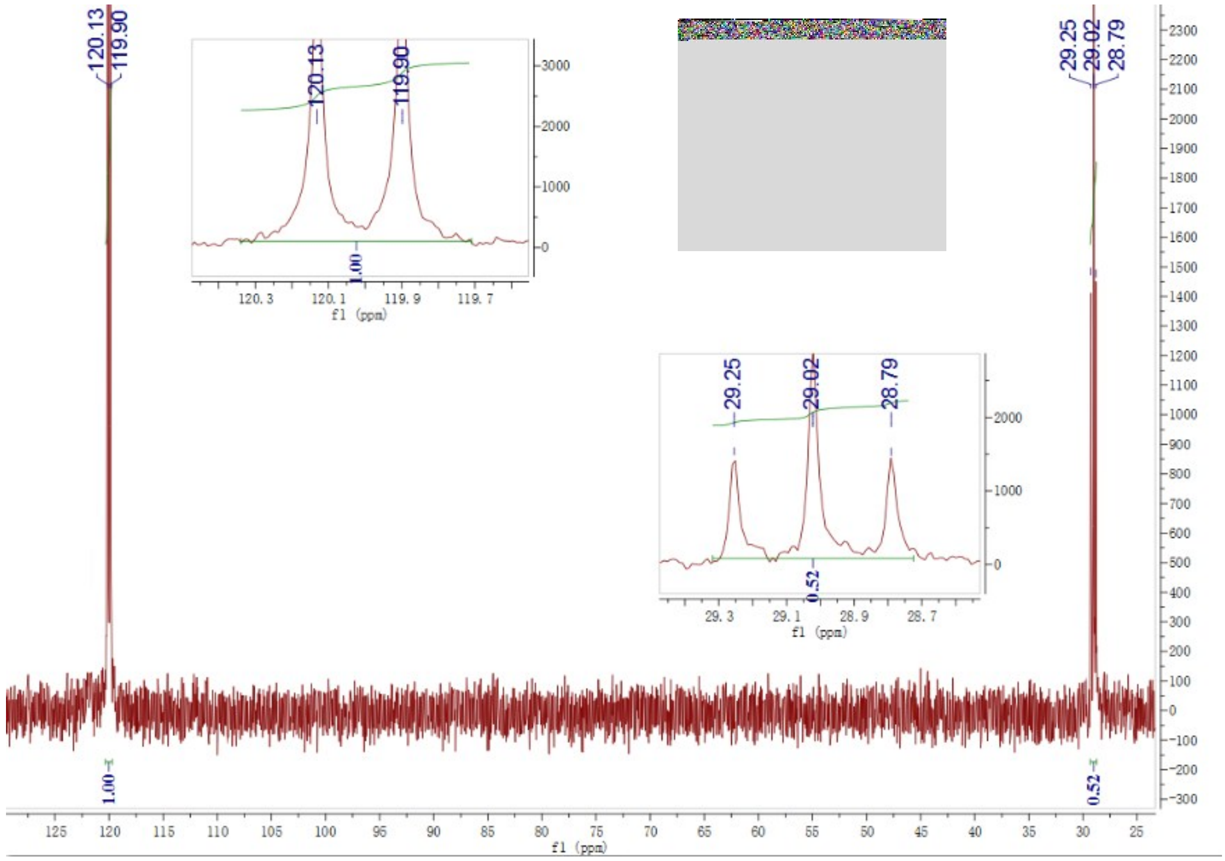

Fig. S8 ^{31}P NMR of complex **6.**

^{13}C NMR of complex **6**

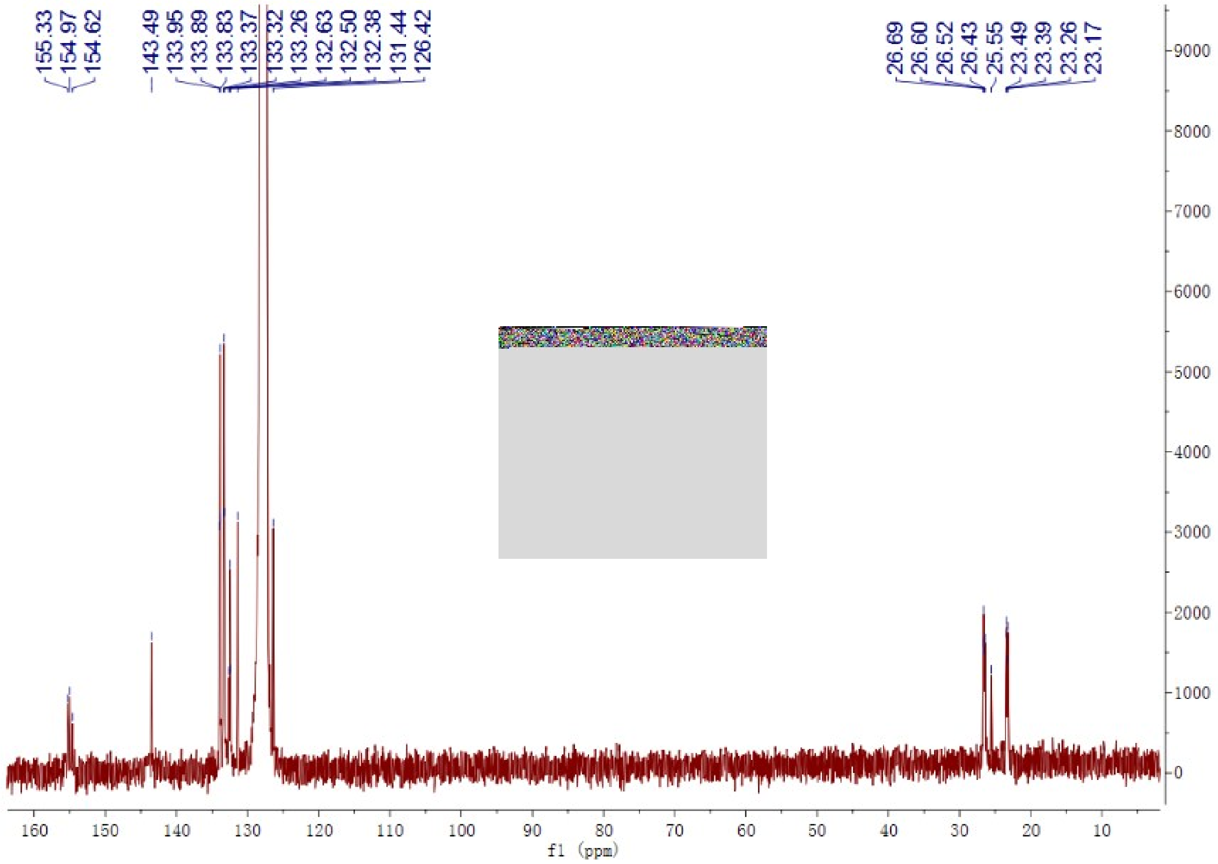

Fig. S9 ^{13}C NMR of complex **6.**

^{29}Si NMR of complex **6**

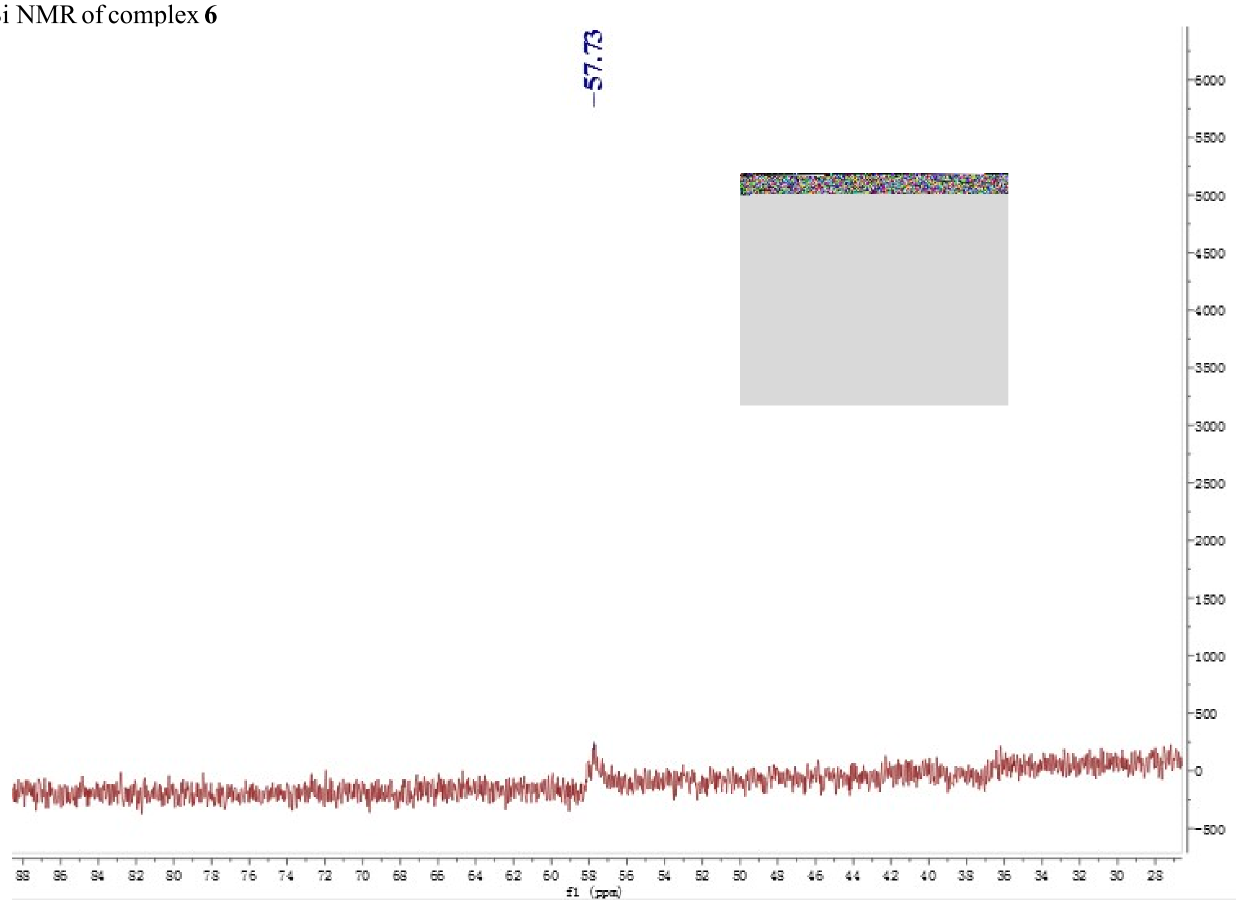

Fig. S10 ^{29}Si NMR of complex **6.**

IR spectrum of complex **7**

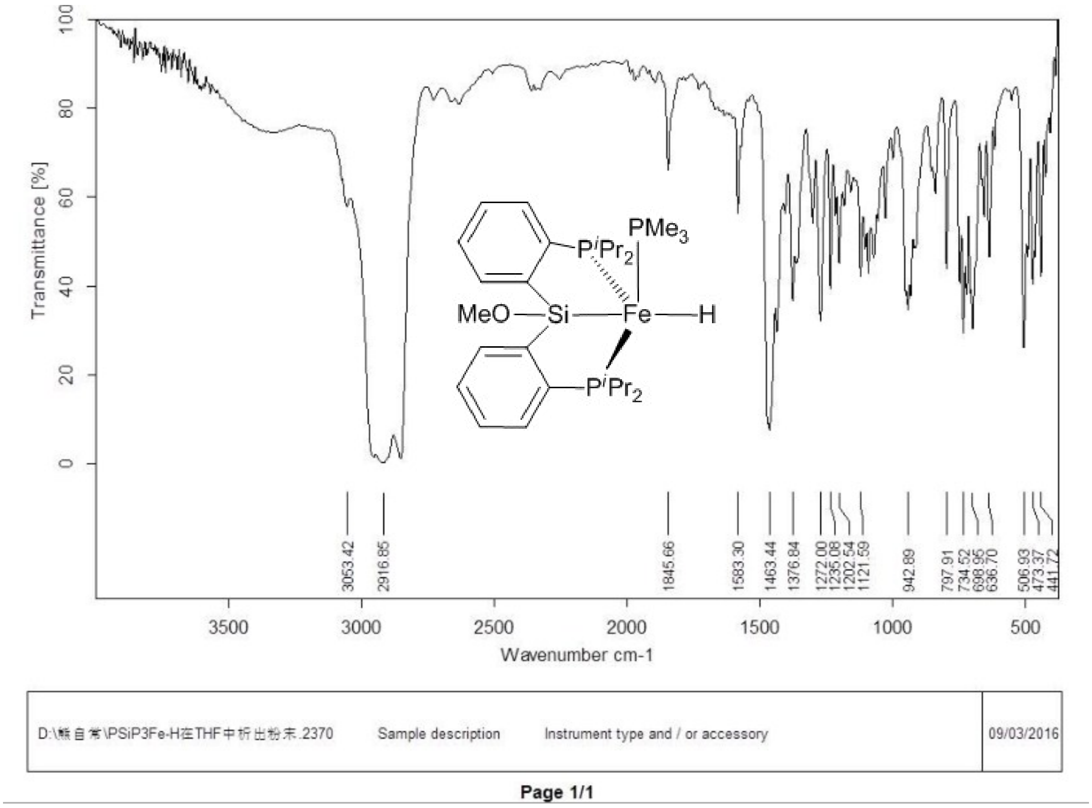

Fig. S11 IR spectrum of complex **7.**

^{1}H NMR of complex **7**

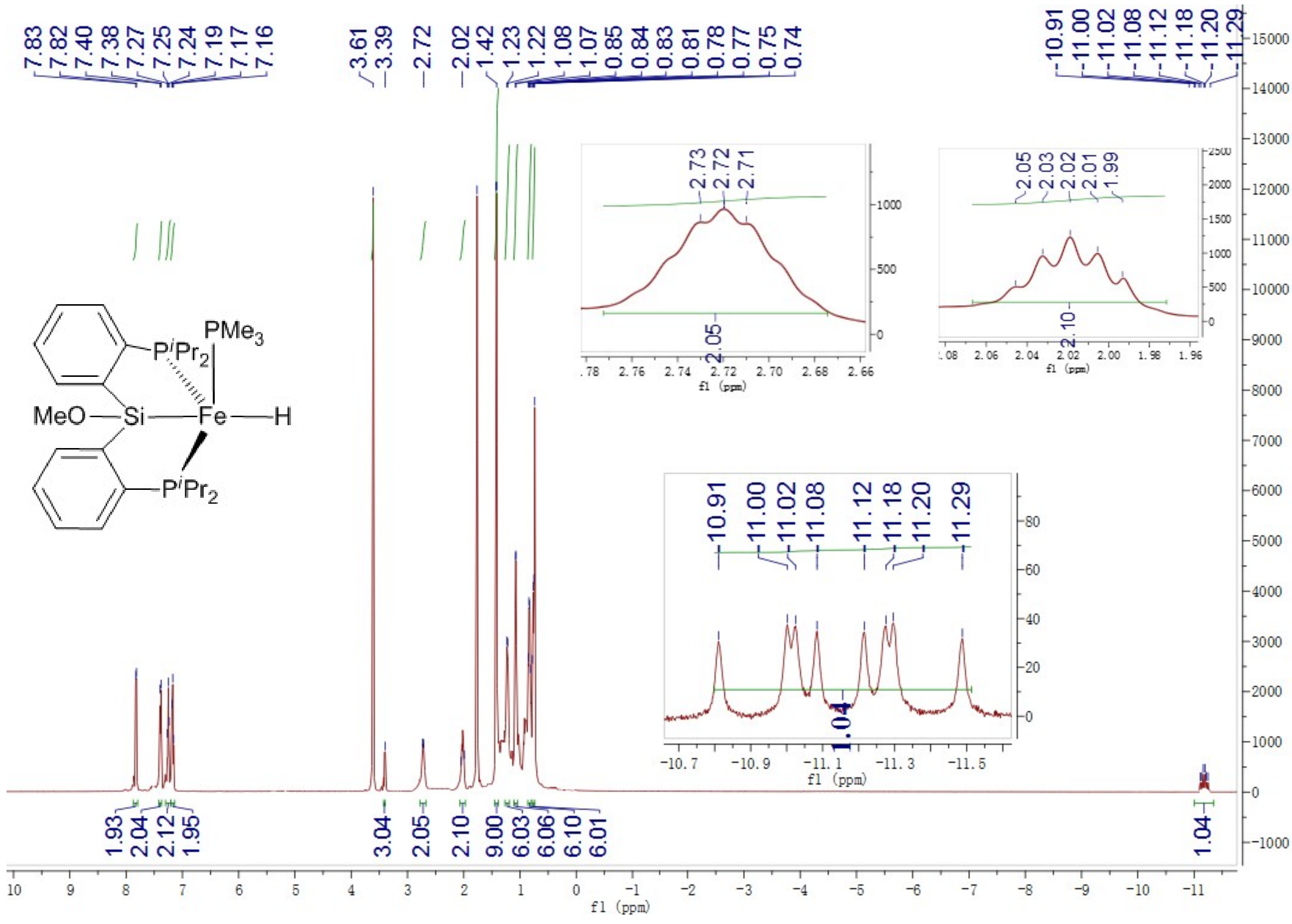

Fig. S12 ^{1}H NMR of complex **7** at -40℃

^{31}P NMR of complex **7**

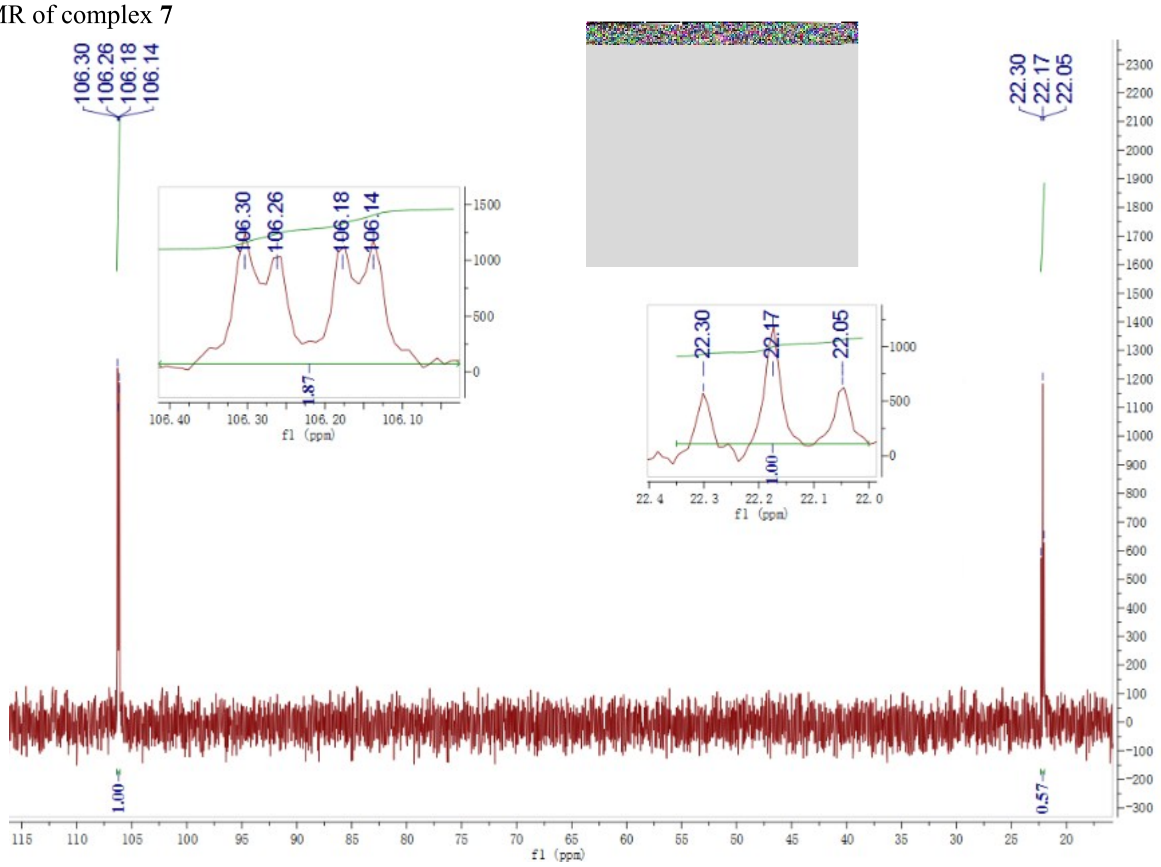

Fig. S13 ^{31}P NMR of complex **7.**

^{13}C NMR of complex **7**

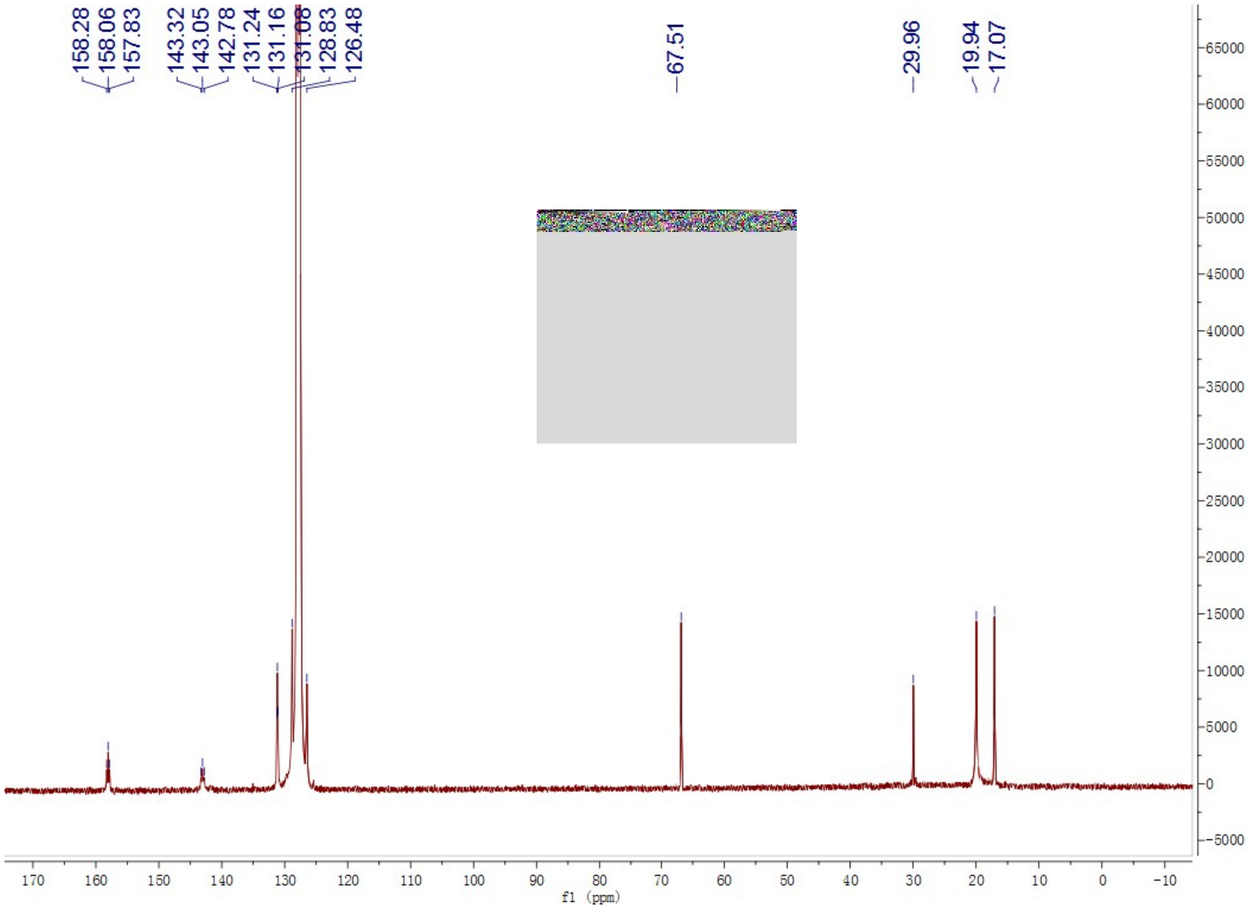

Fig. S14 ^{13}C NMR of complex **7.**

^{29}Si NMR of complex **7**

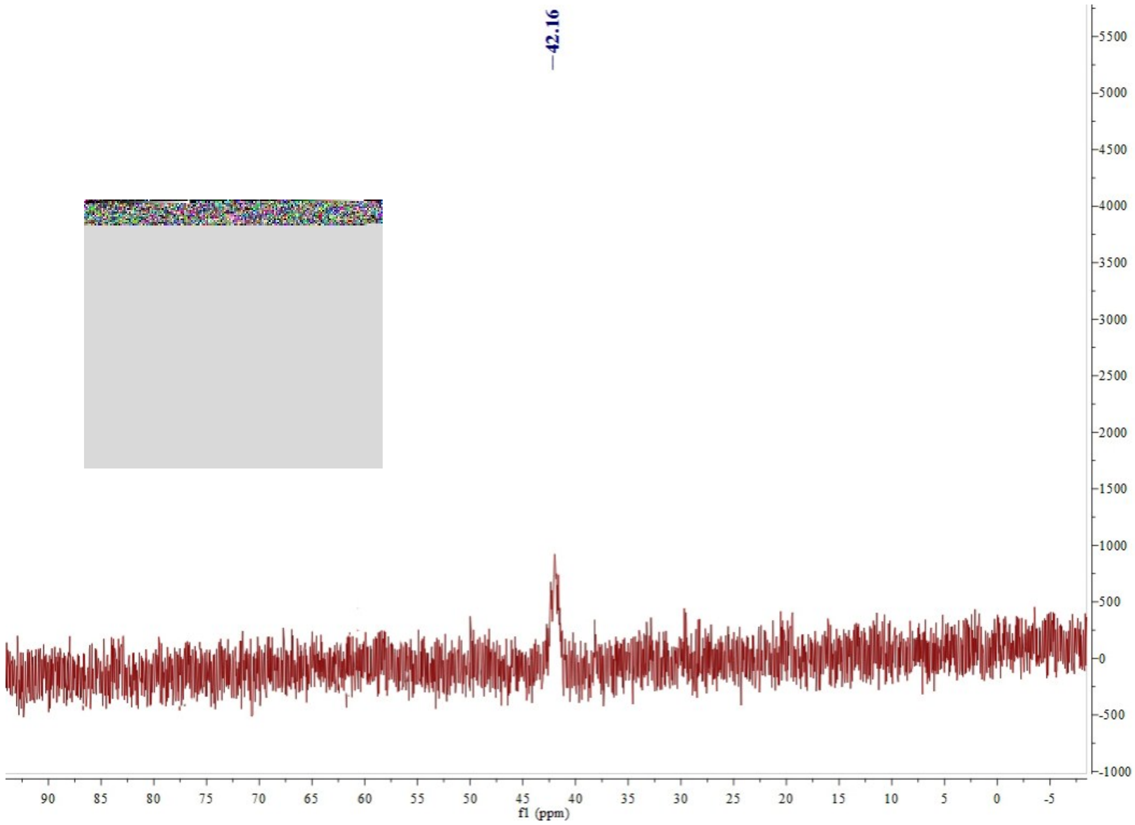

Fig. S15 ^{29}Si NMR of complex **7.**

3 NMR spectra of the catalytic products

^{1}H NMR of compound 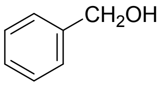

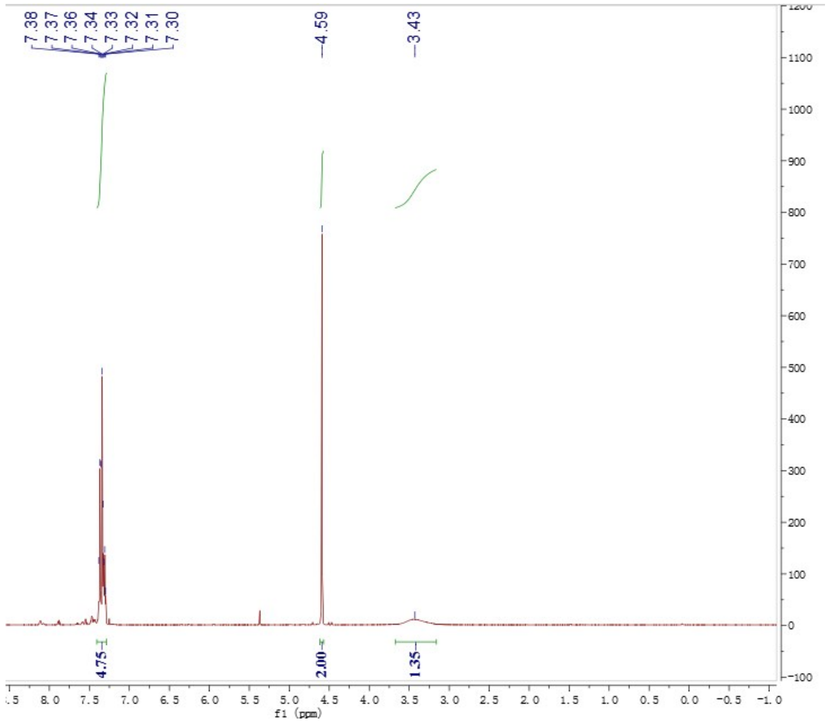

^{13}C NMR of compound 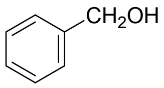

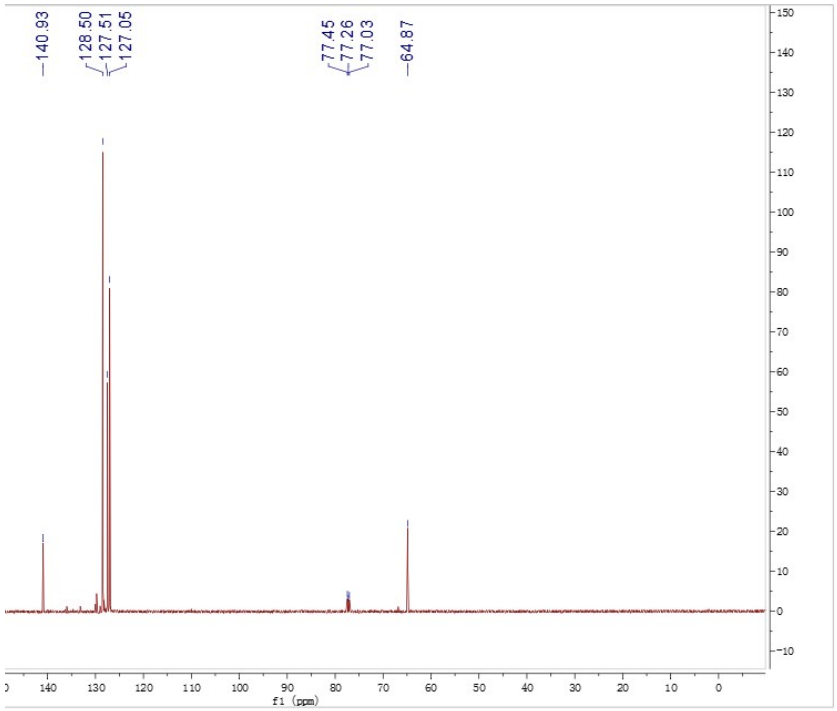

^{1}H NMR of compound 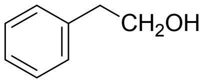

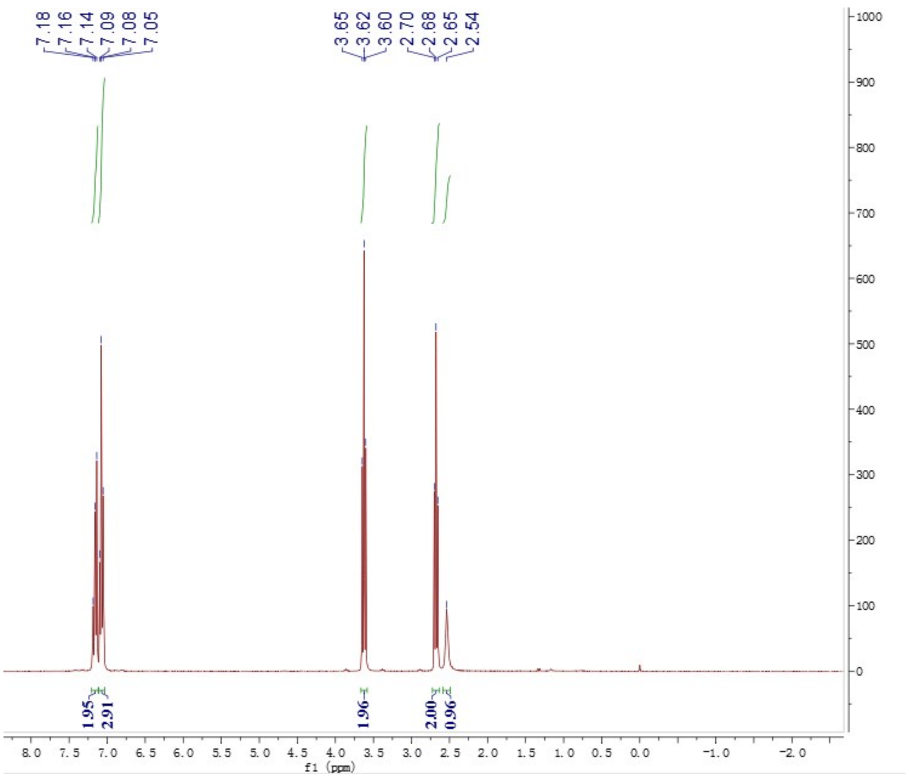

^{13}C NMR of compound 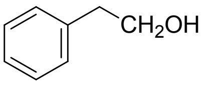

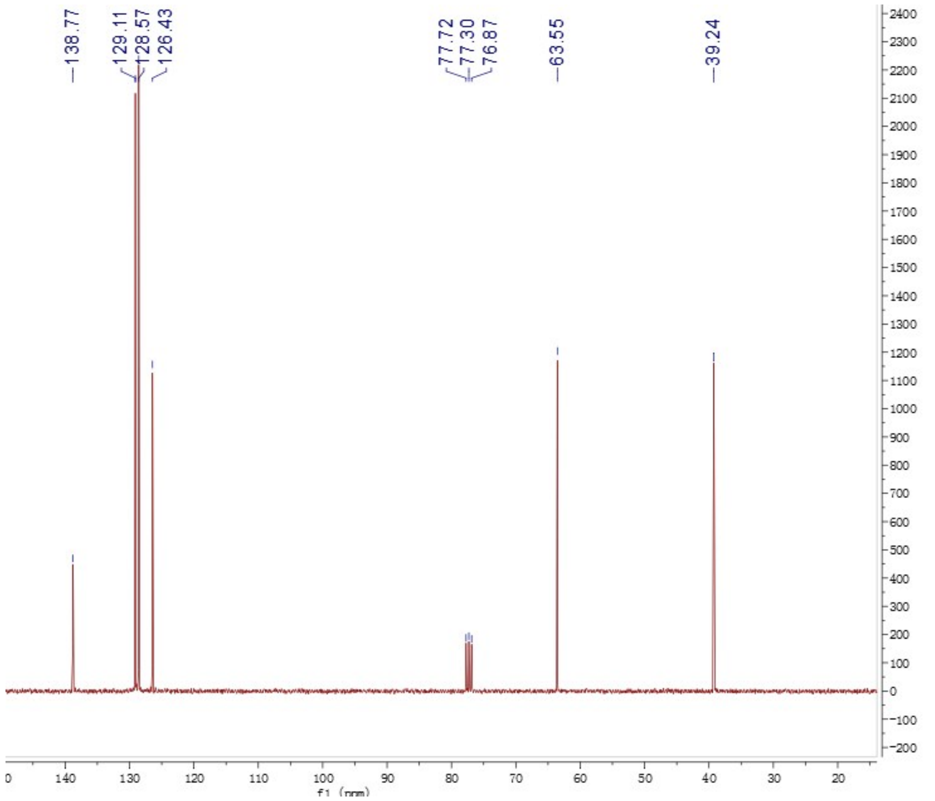

^{1}H NMR of compound 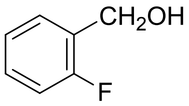

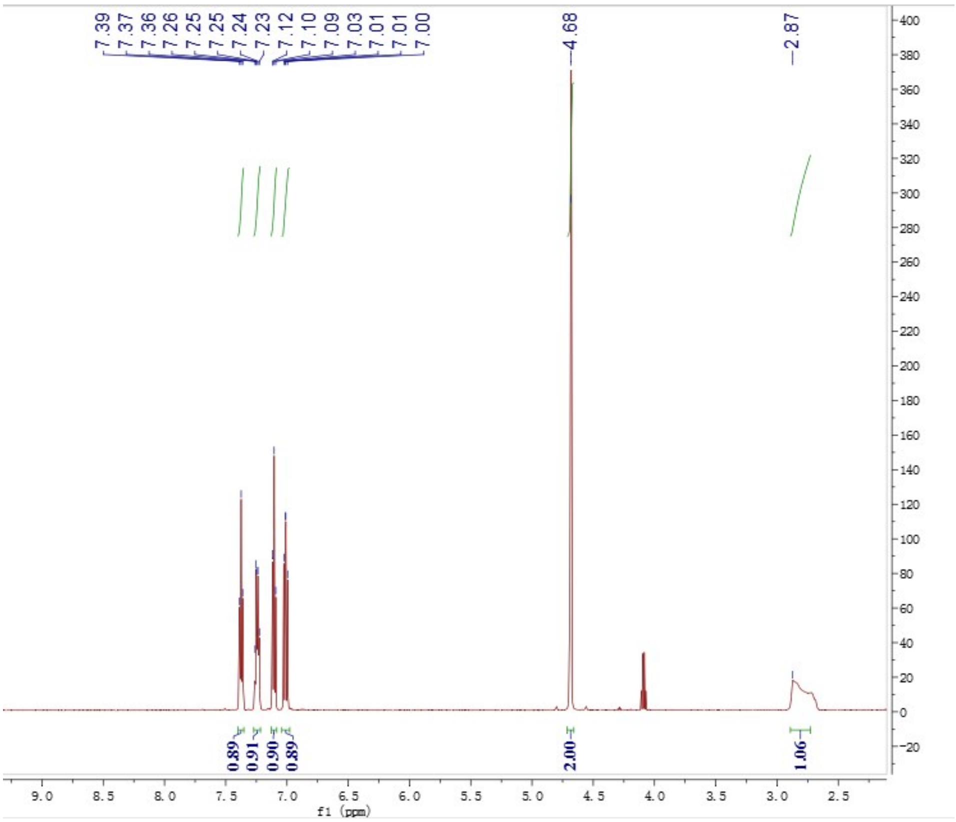

^{13}C NMR of compound 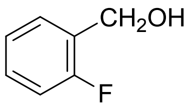

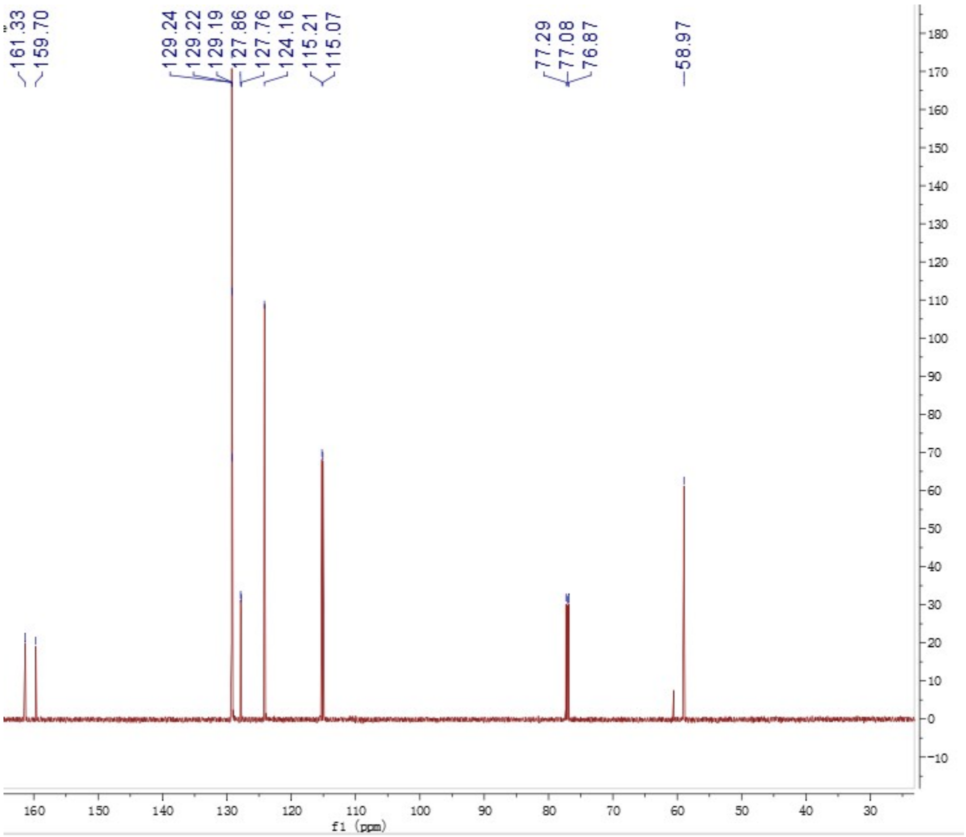

^{1}H NMR of compound 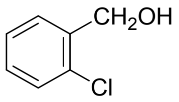

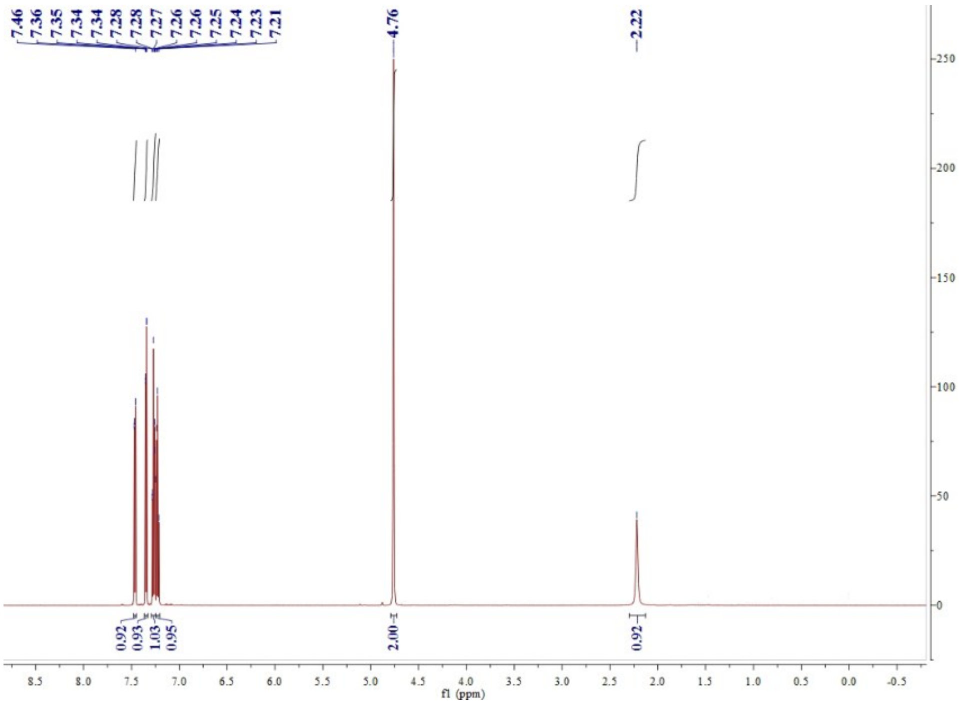

^{13}C NMR of compound 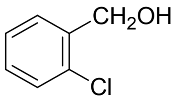

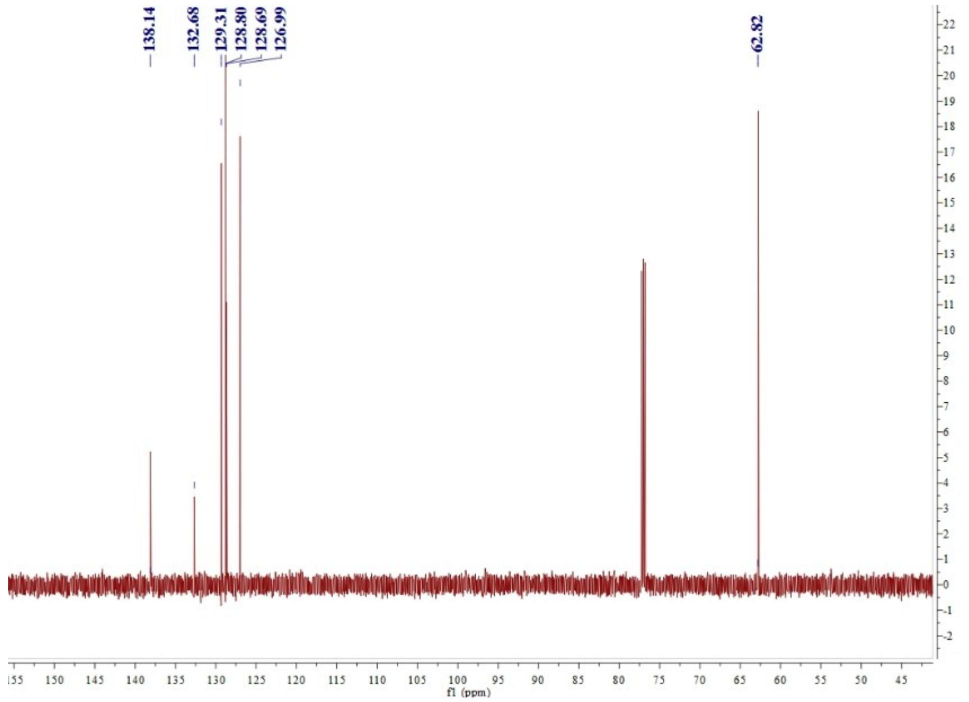

^{1}H NMR of compound 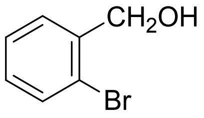

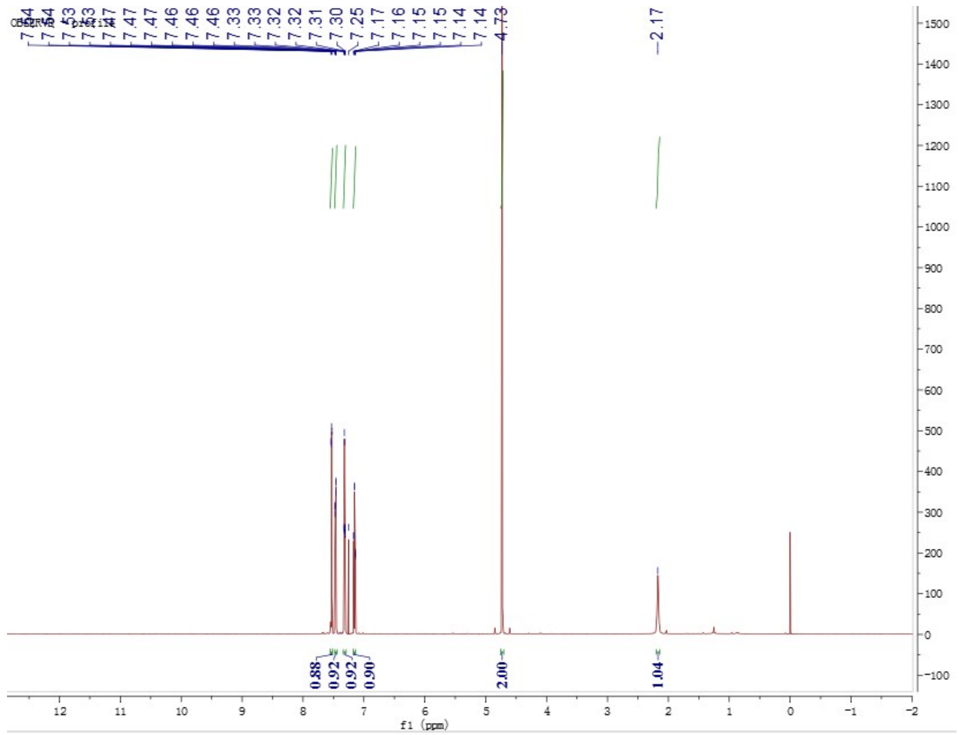

^{13}C NMR of compound 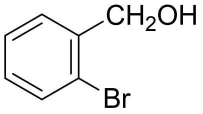

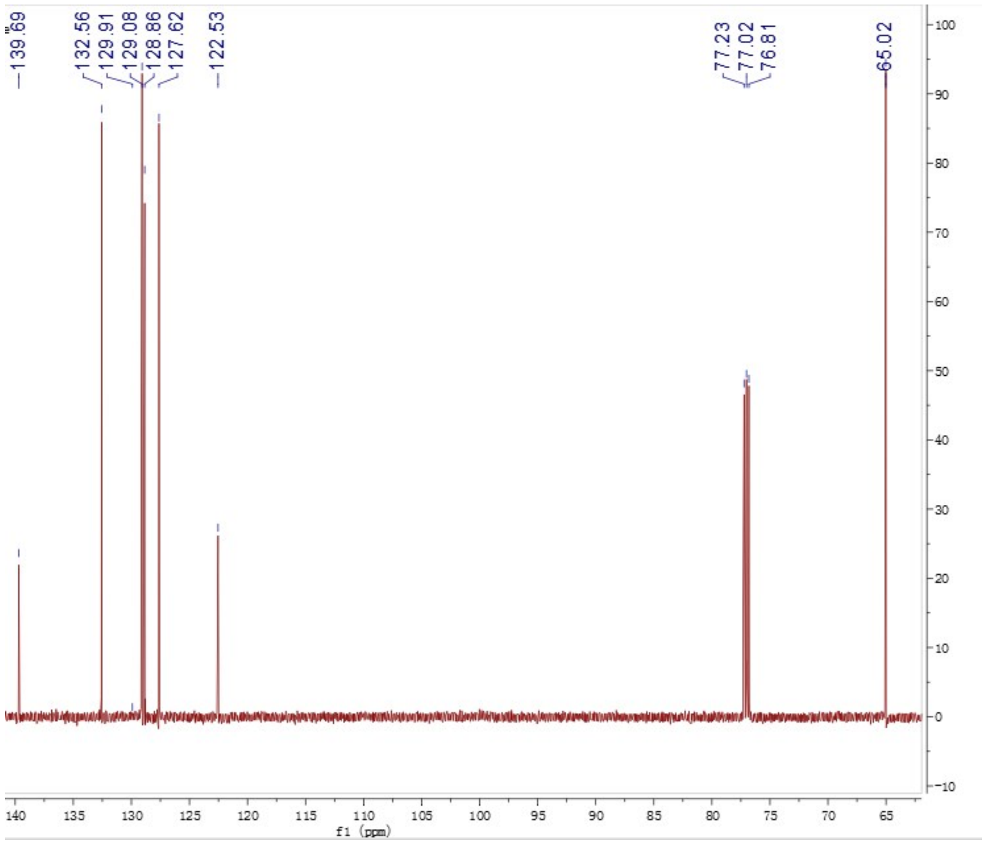

^{1}H NMR of compound 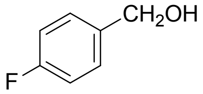

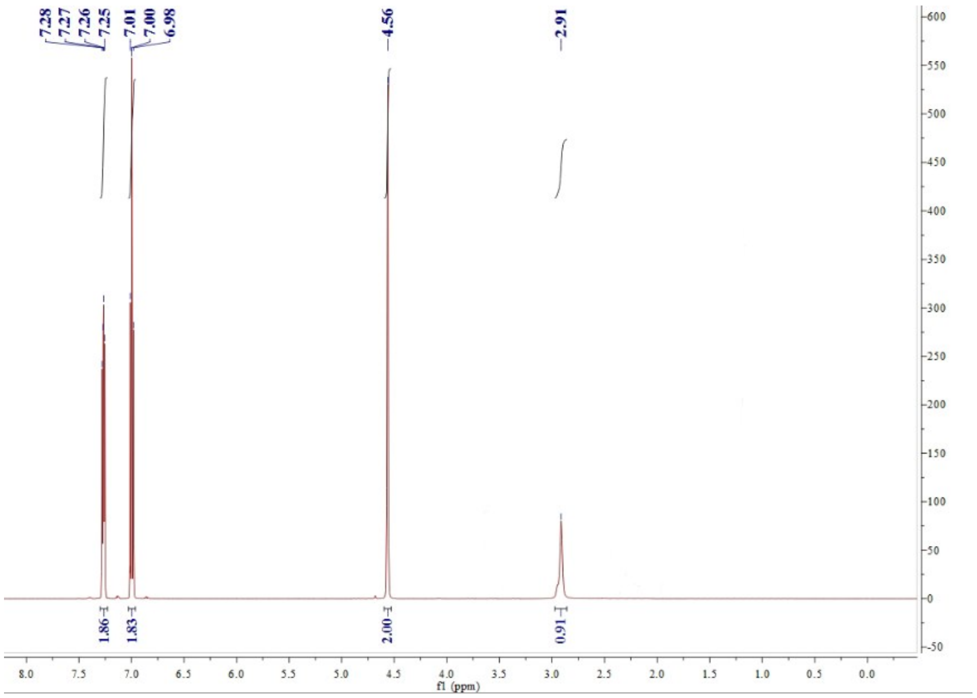

^{13}C NMR of compound 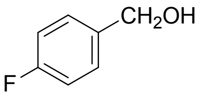

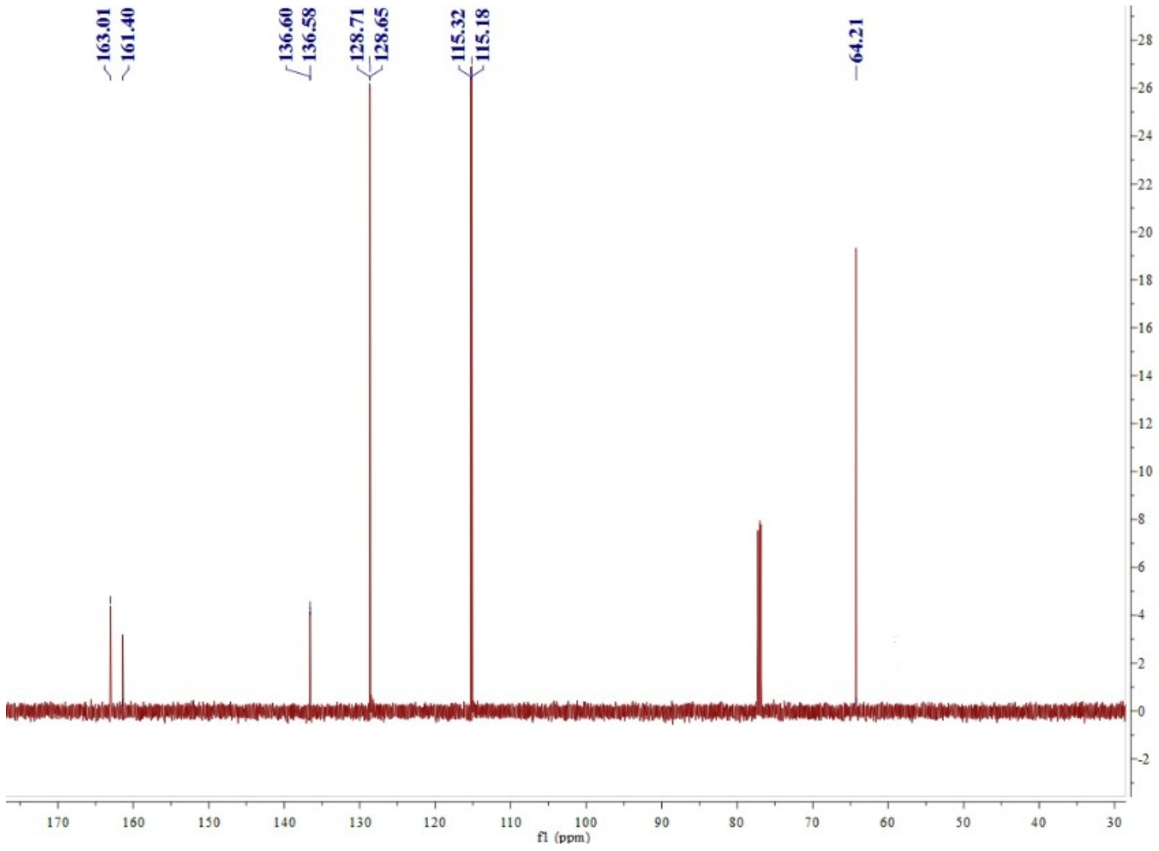

^{1}H NMR of compound 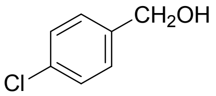

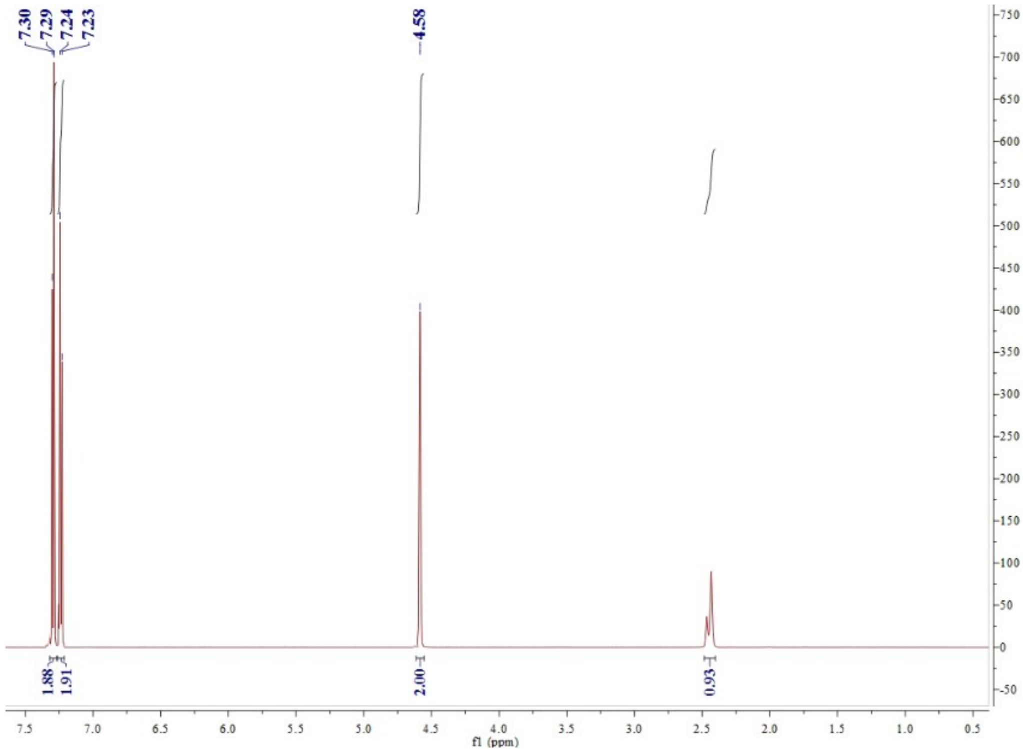

^{13}C NMR of compound 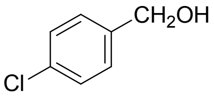

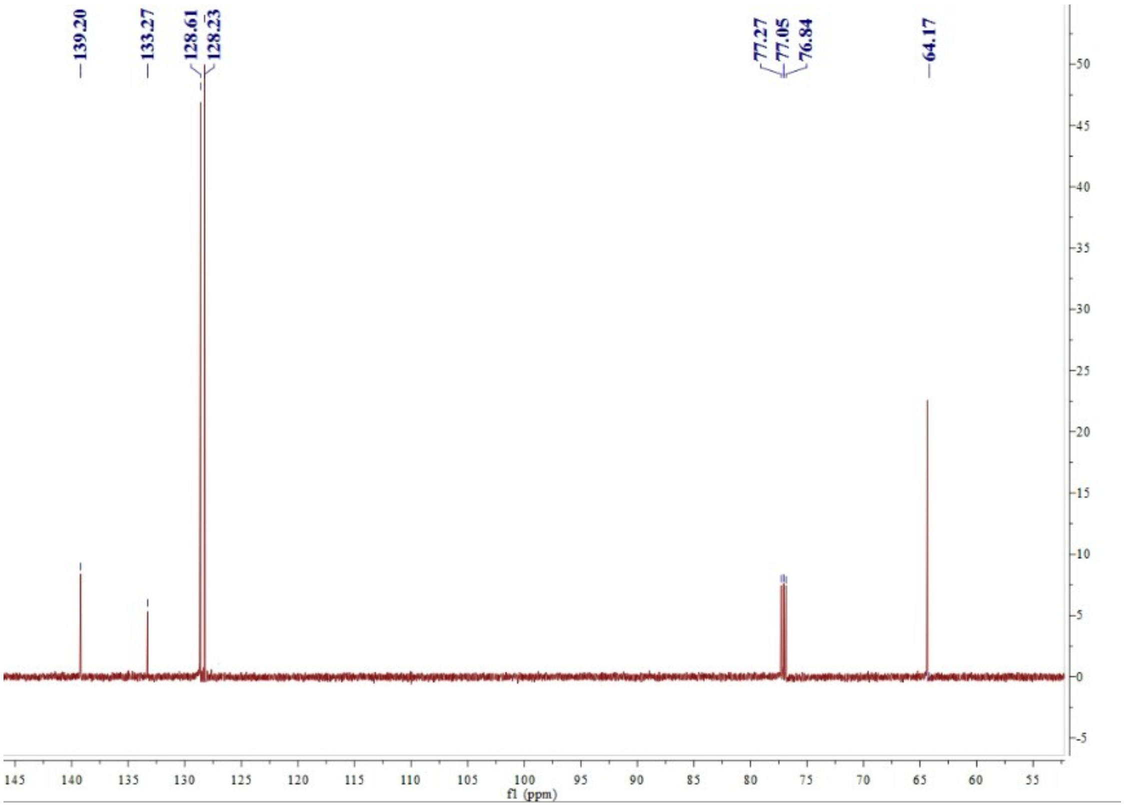

^{1}H NMR of compound 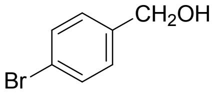

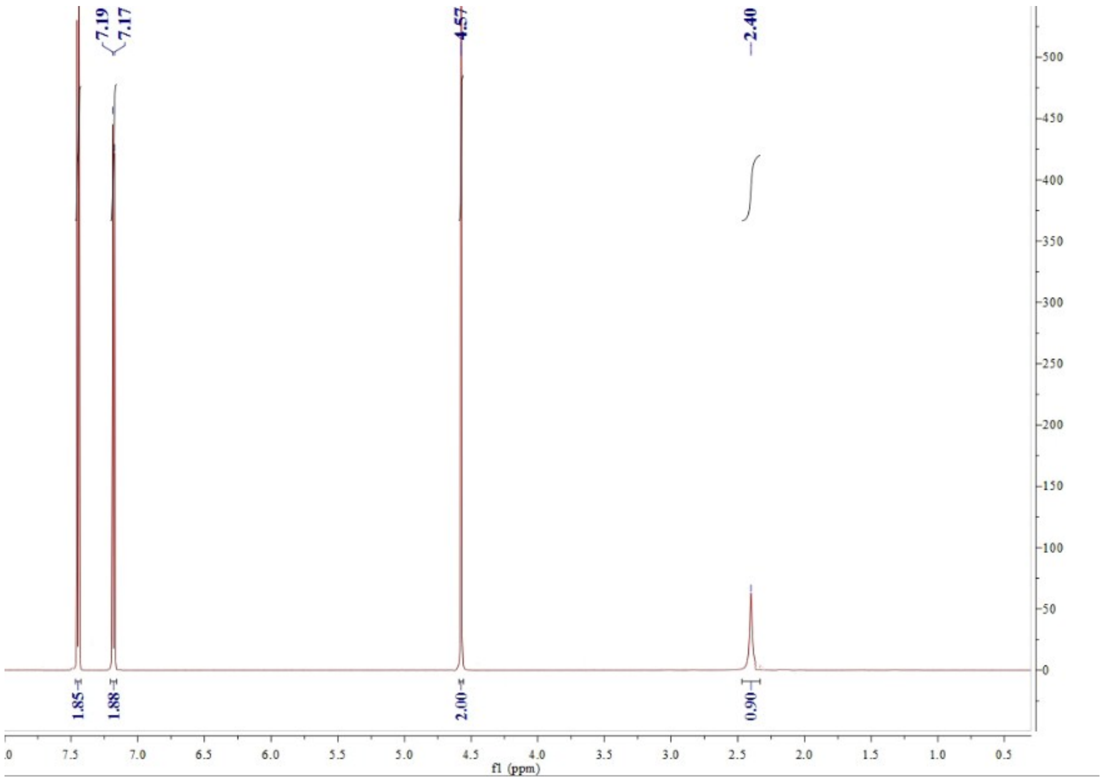

^{13}C NMR of compound 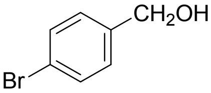

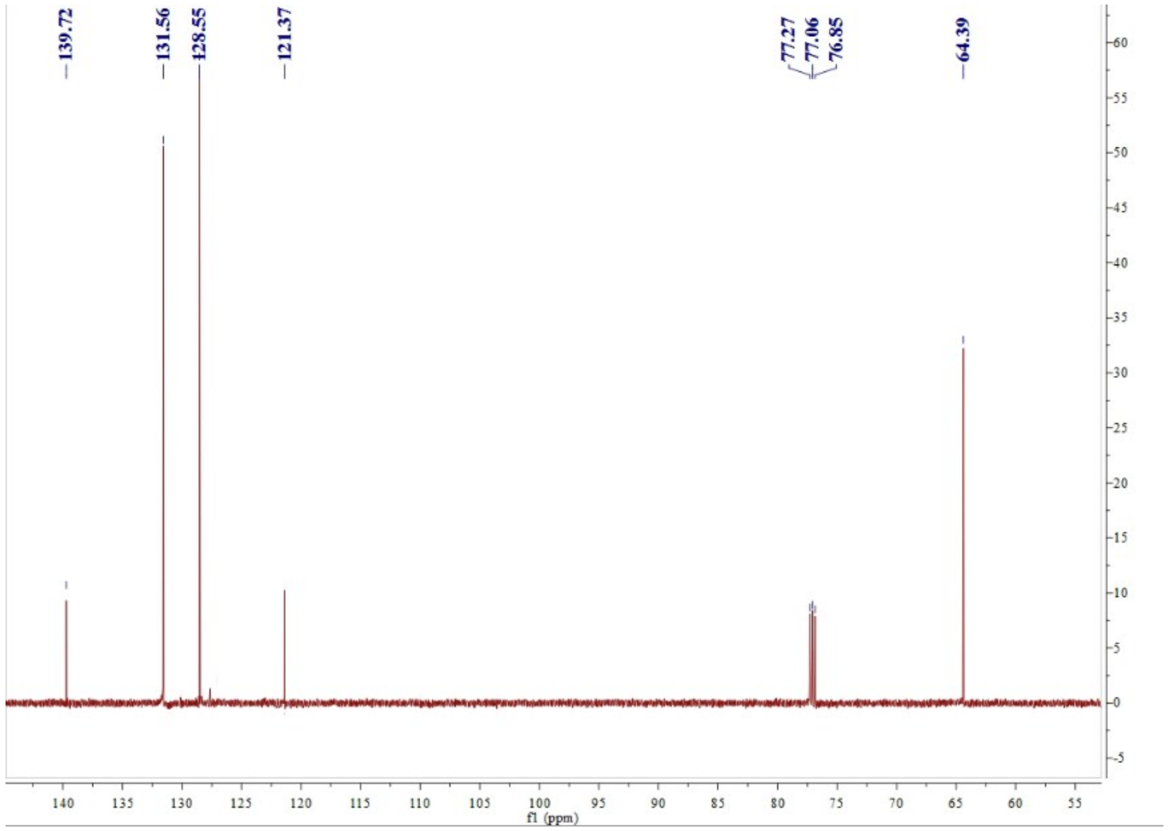

^{1}H NMR of compound 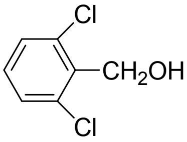

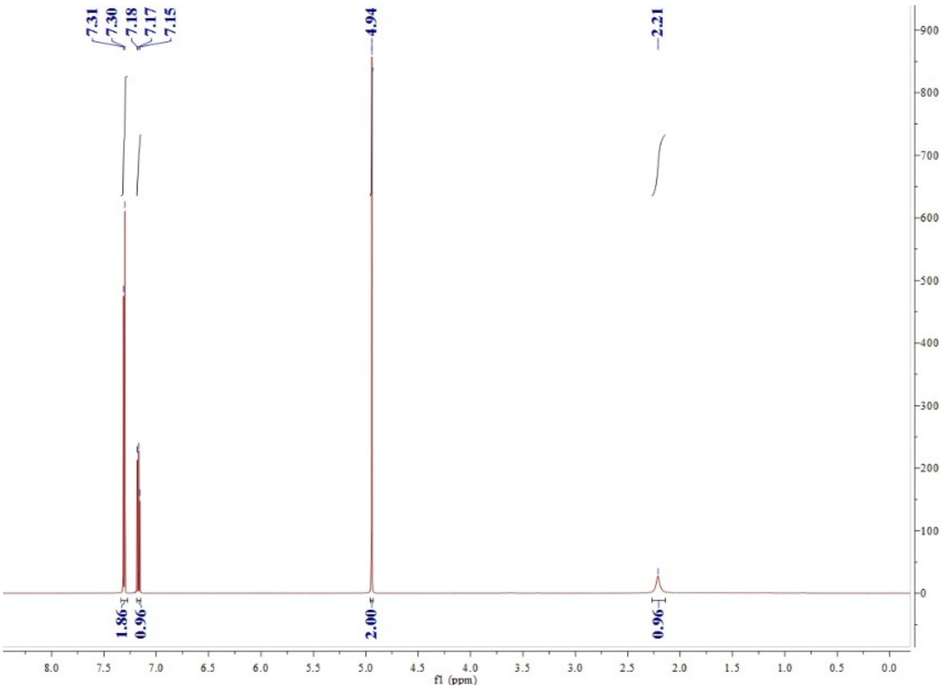

^{13}C NMR of compound 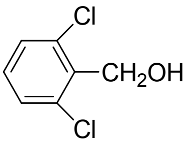

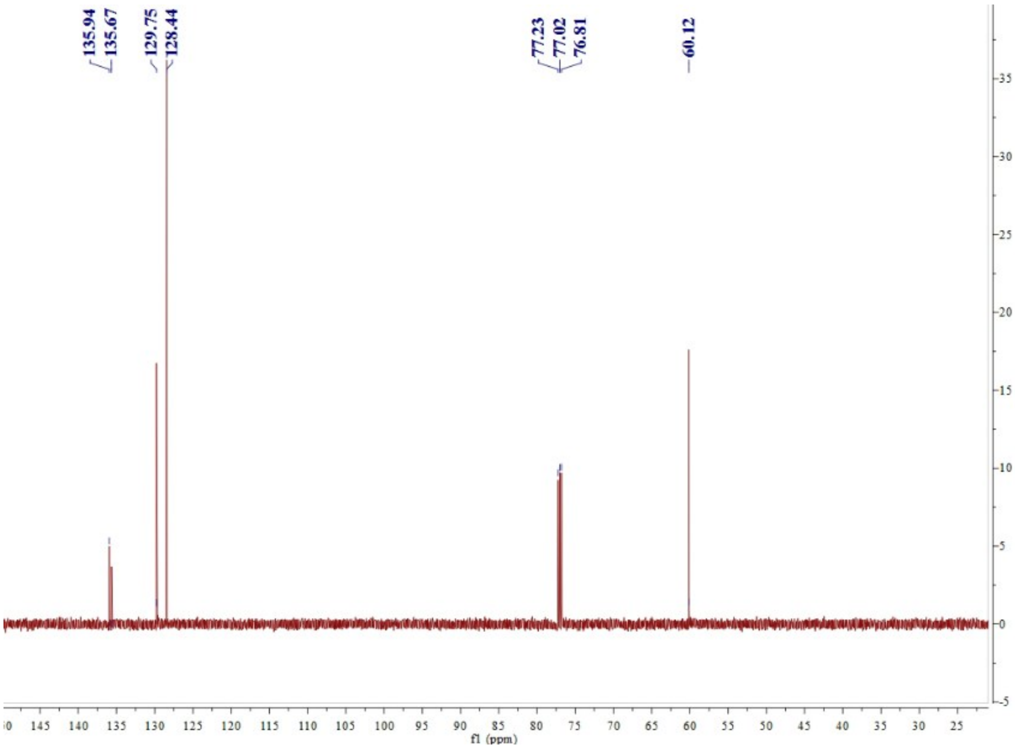

^{1}H NMR of compound 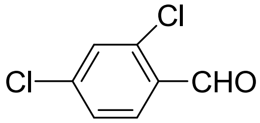

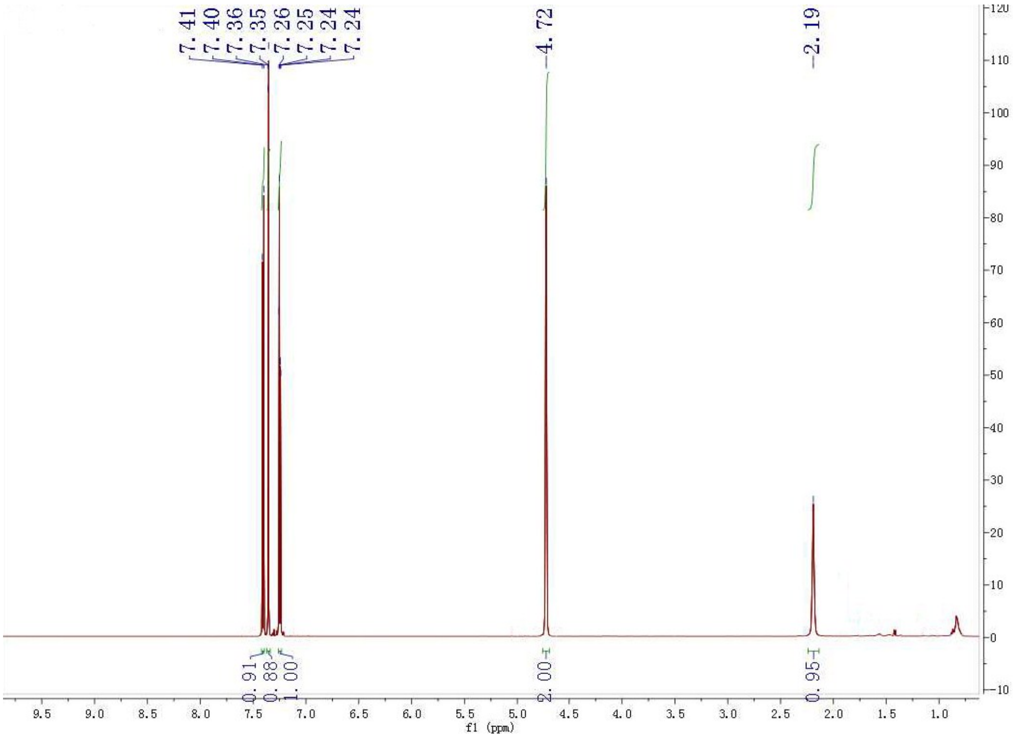

^{13}C NMR of compound 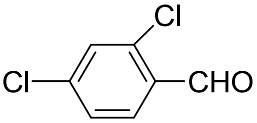

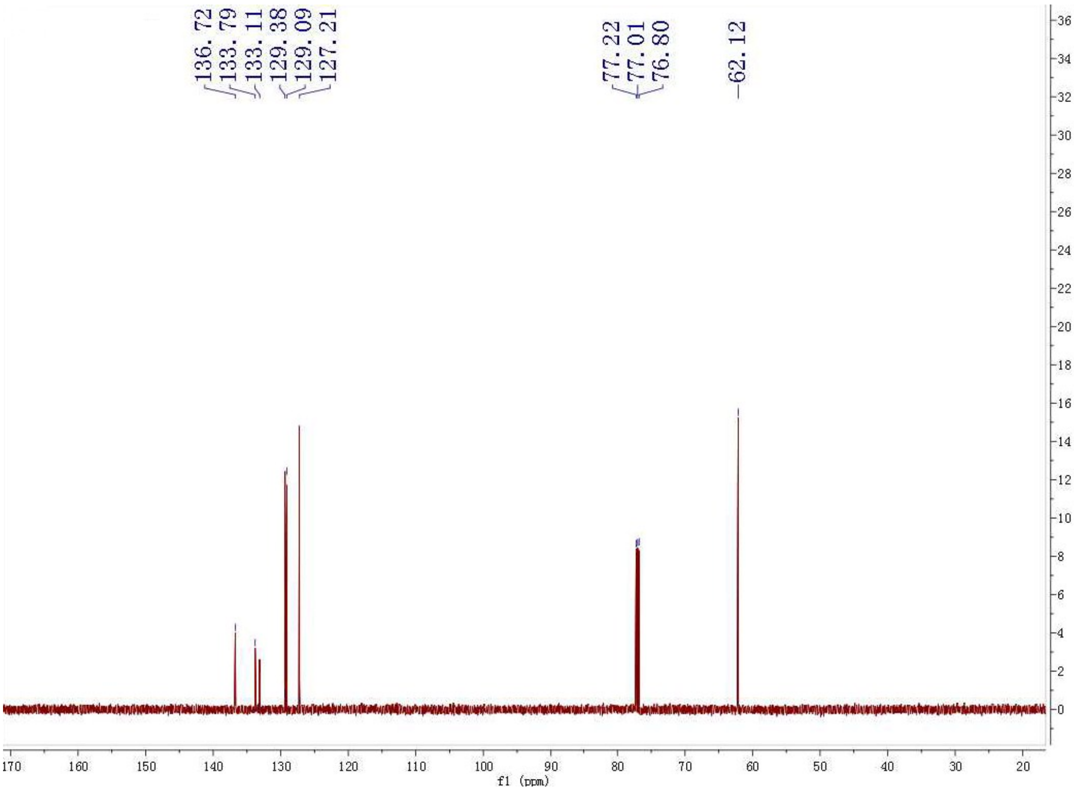

^{1}H NMR of compound 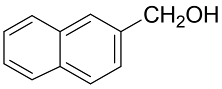

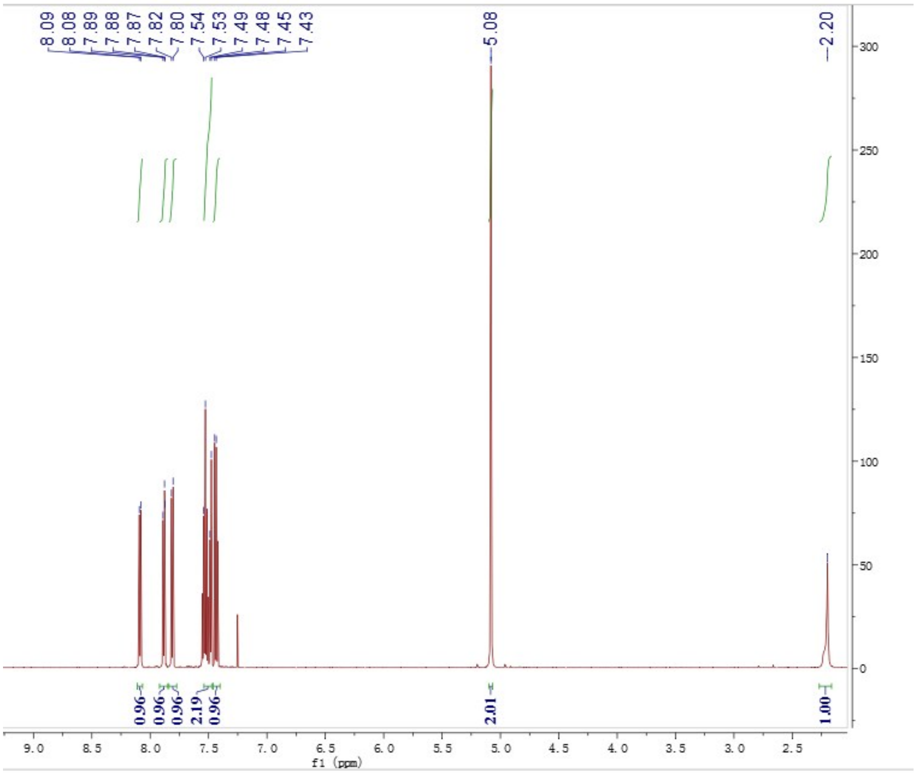

^{13}C NMR of compound 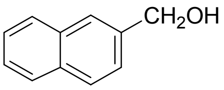

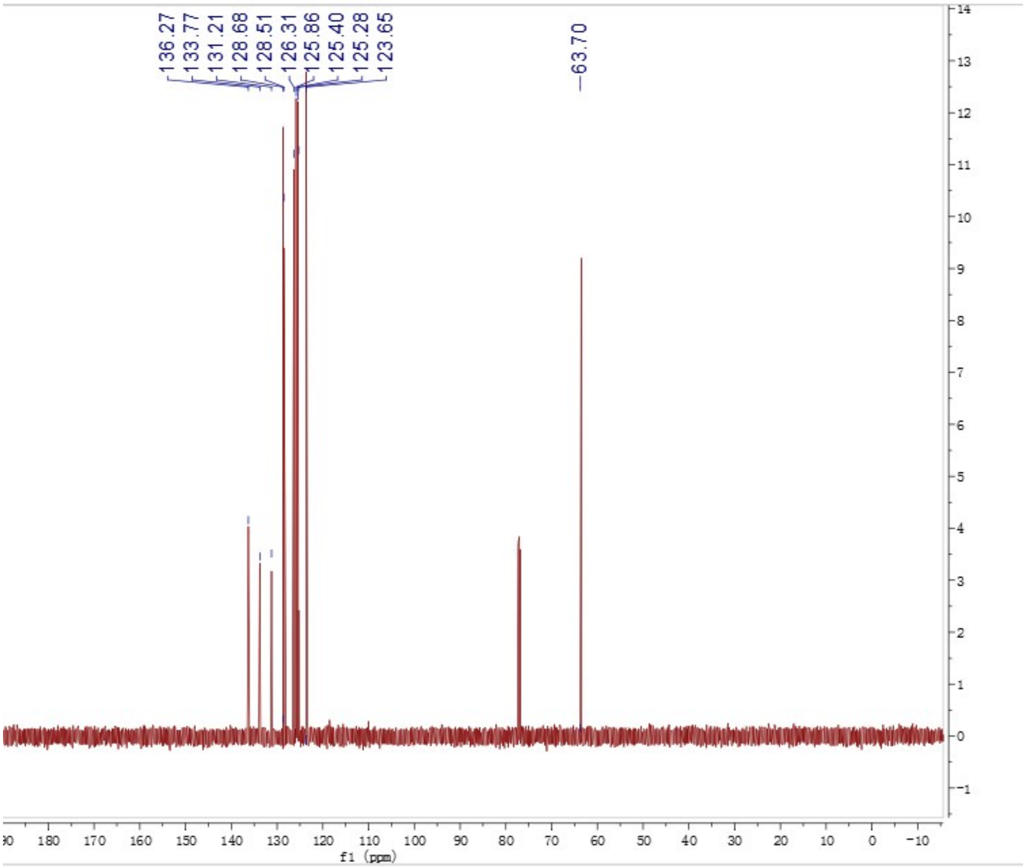

^{1}H NMR of compound 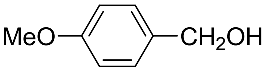

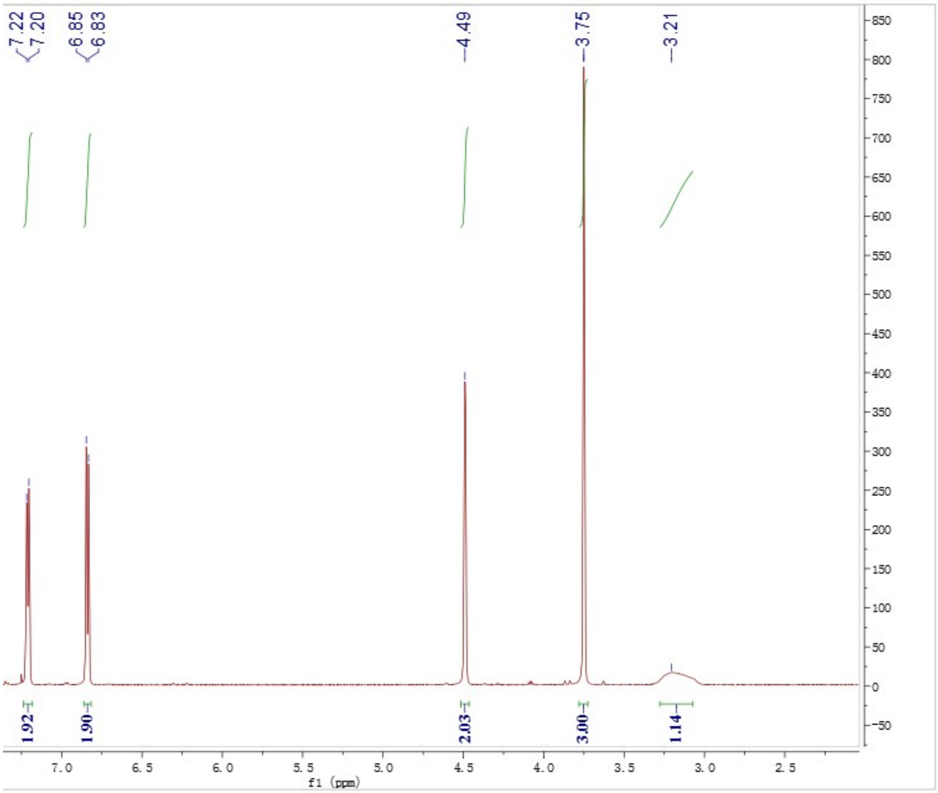

^{13}C NMR of compound 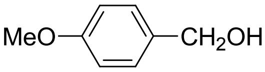

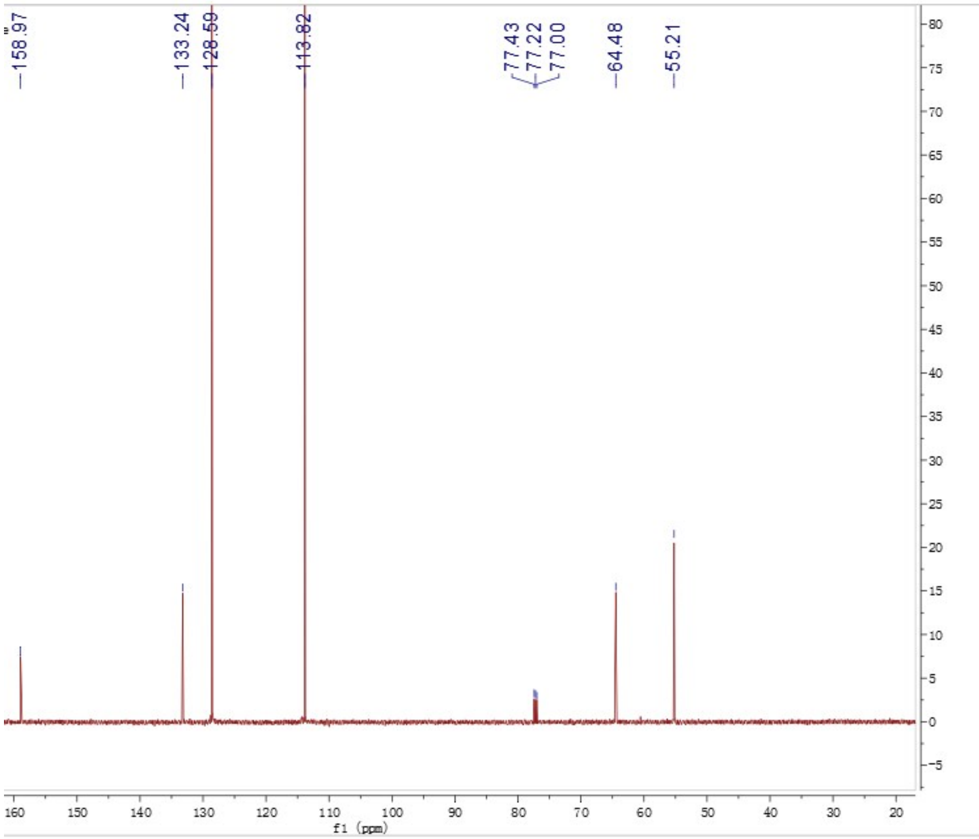

^{1}H NMR of compound 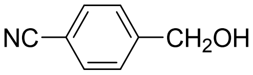

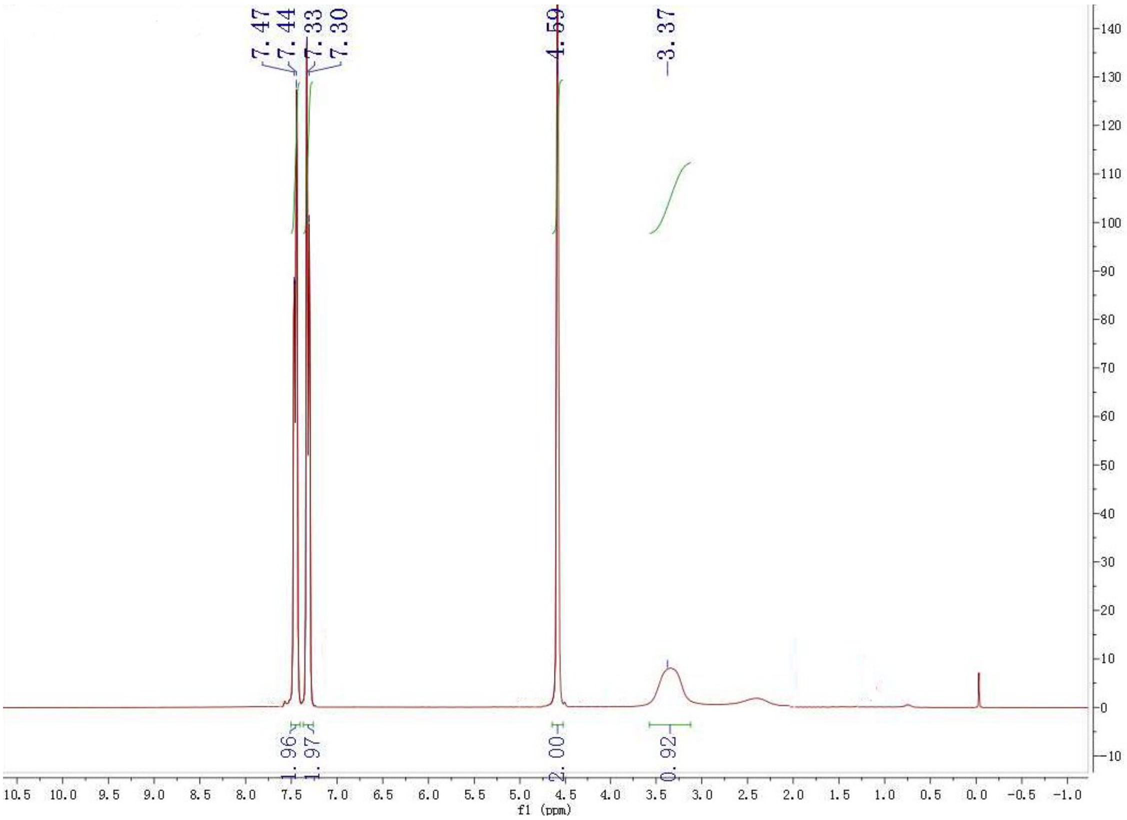

^{13}C NMR of compound 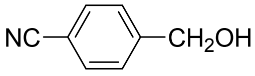

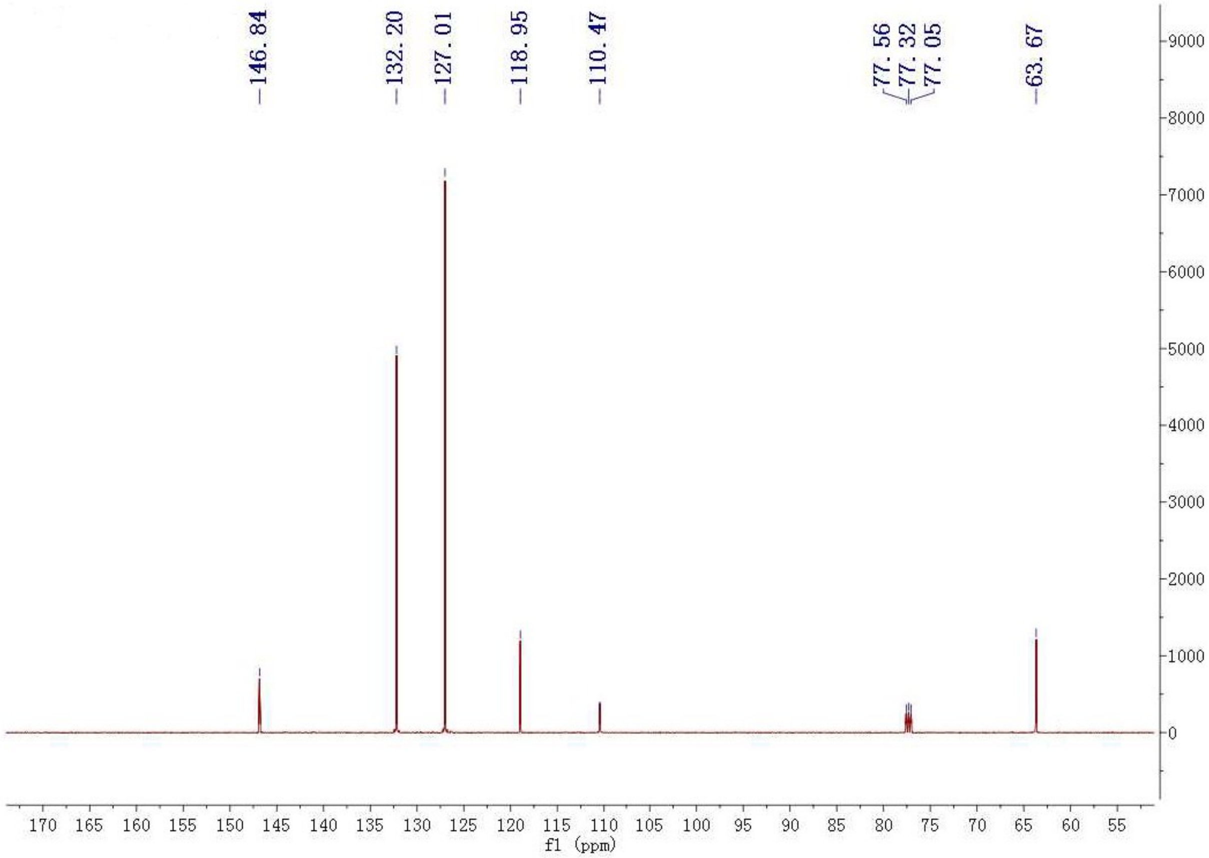

^{1}H NMR of compound 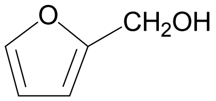

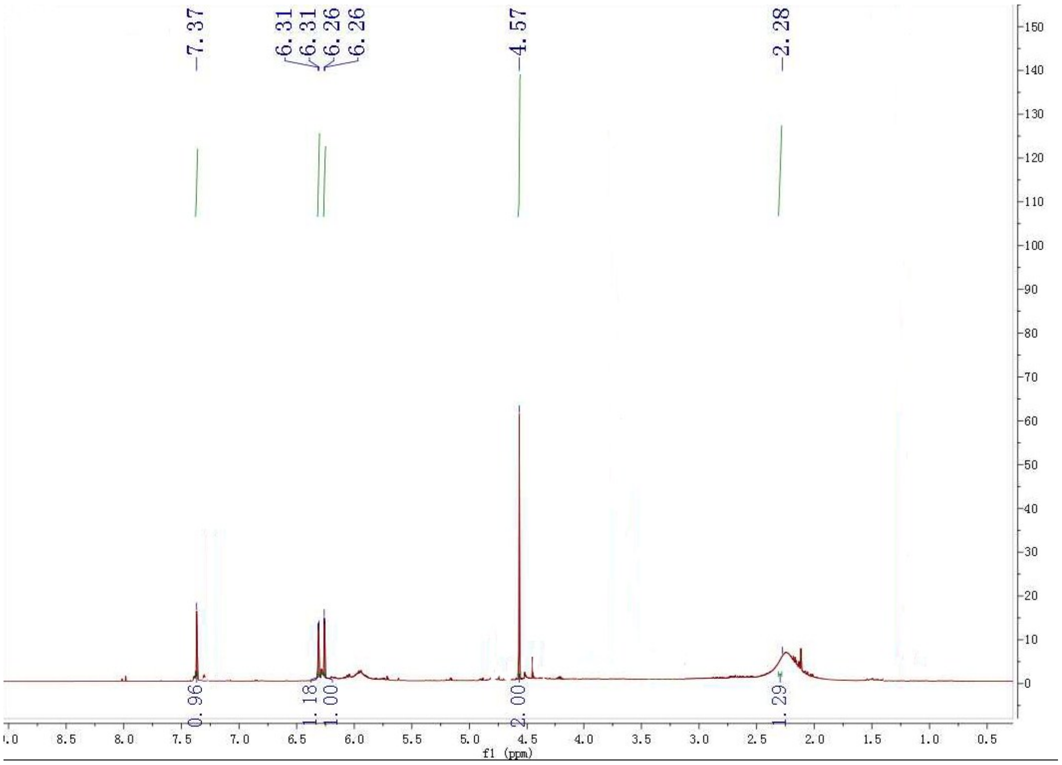

^{13}C NMR of compound 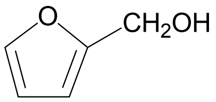

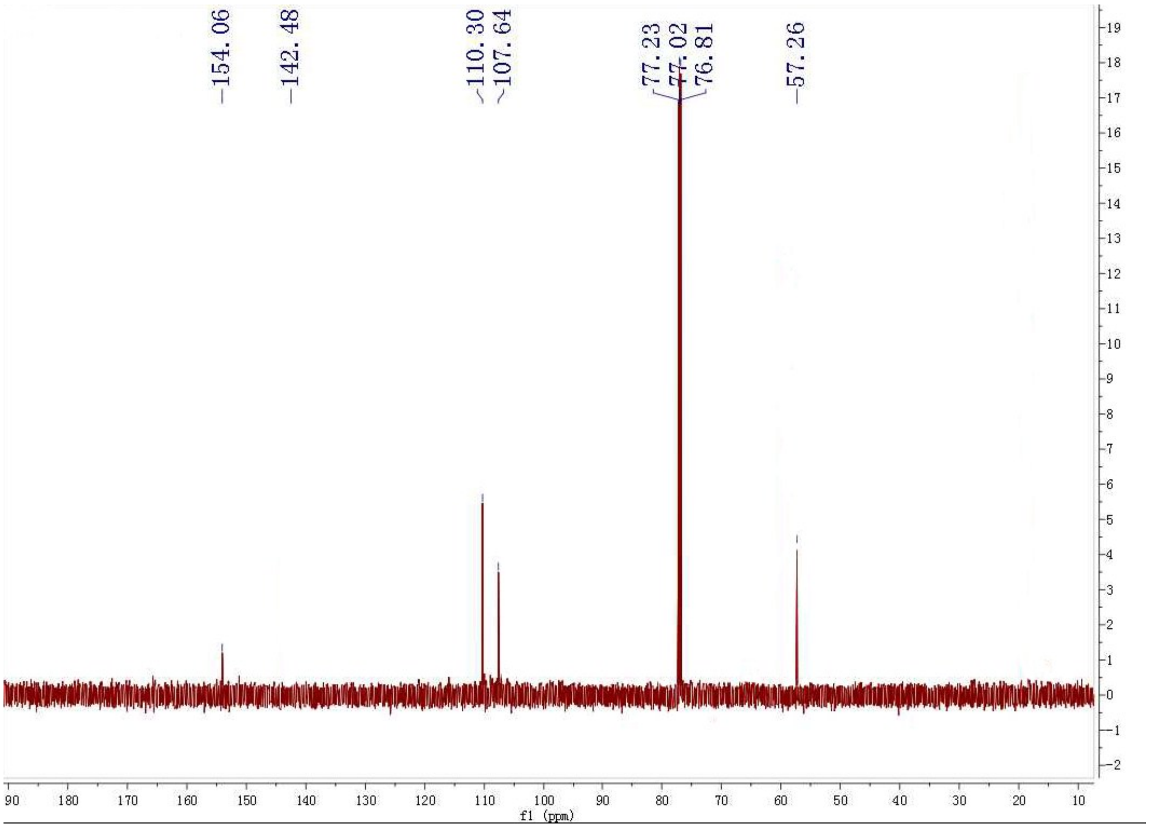

^{1}H NMR of compound 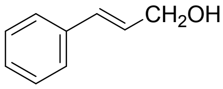

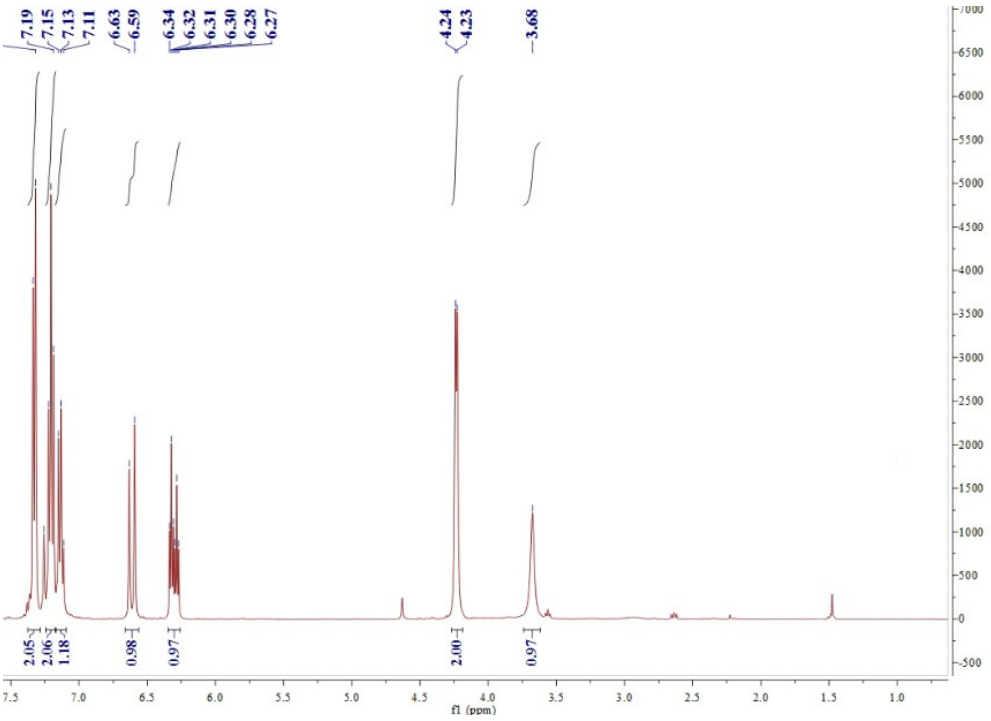

^{13}C NMR of compound 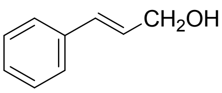

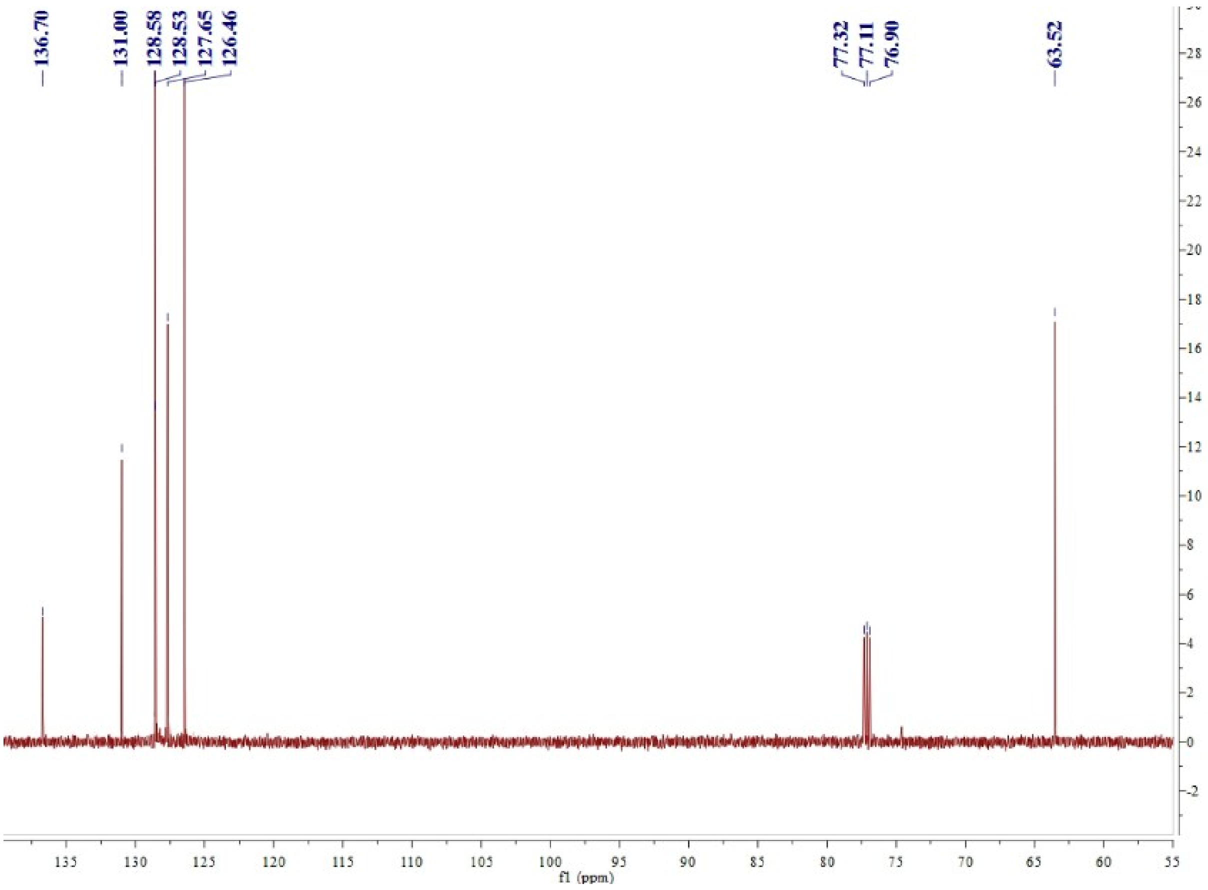

^{1}H NMR of compound 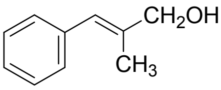

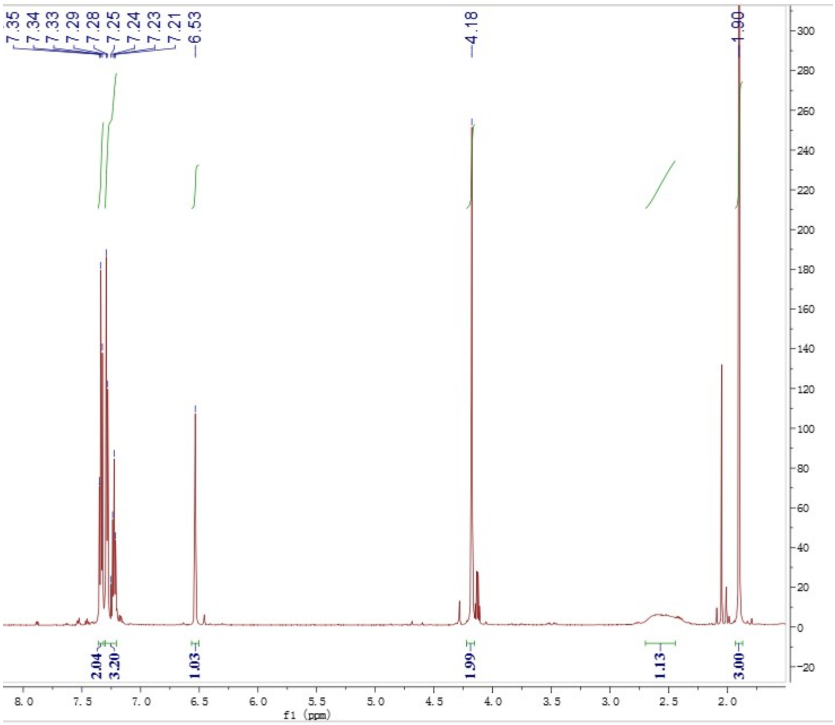

^{13}C NMR of compound 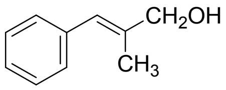

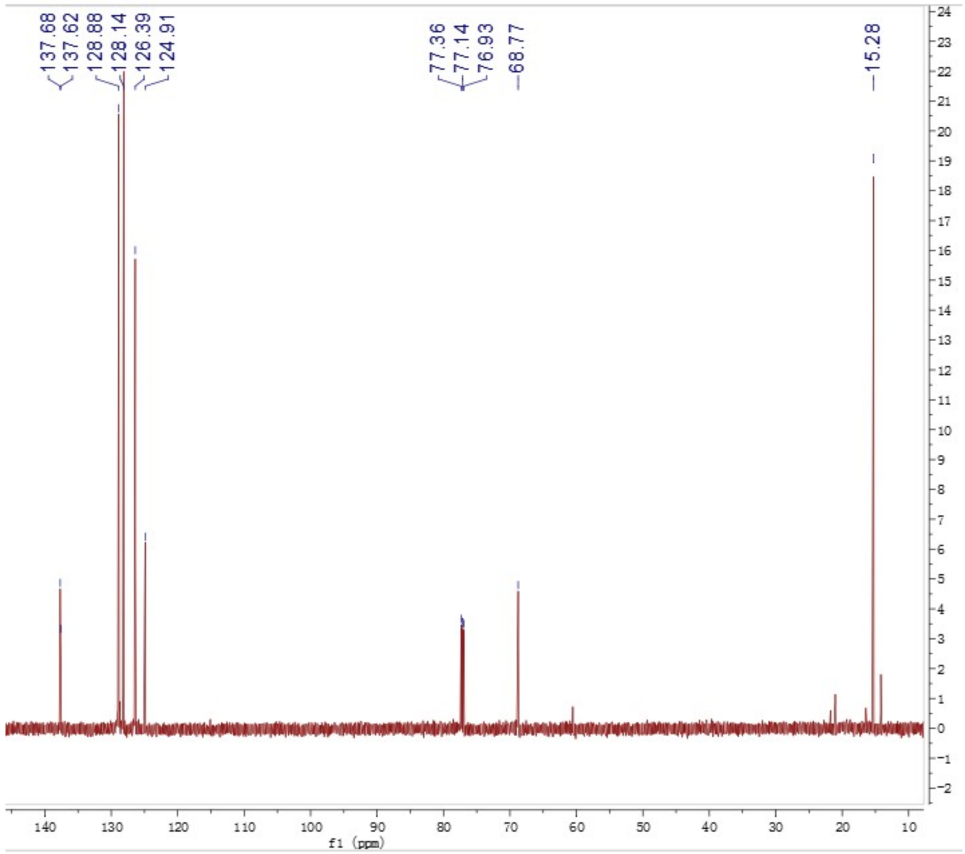

^{1}H NMR of compound 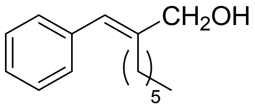

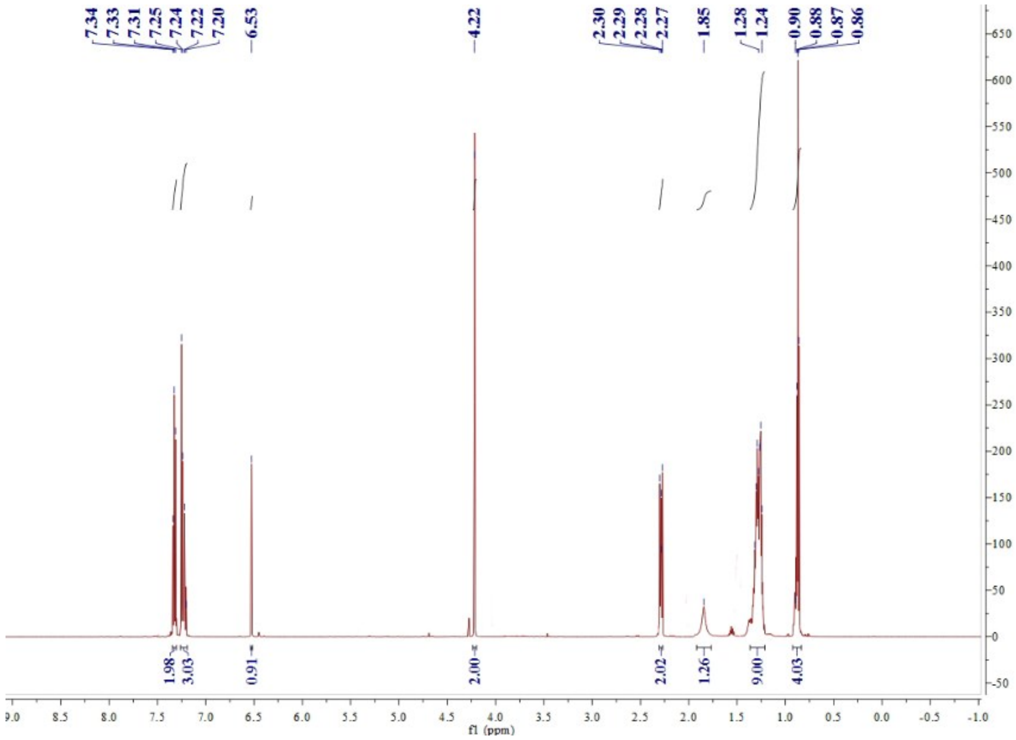

^{13}C NMR of compound 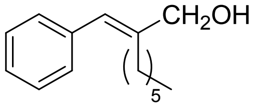

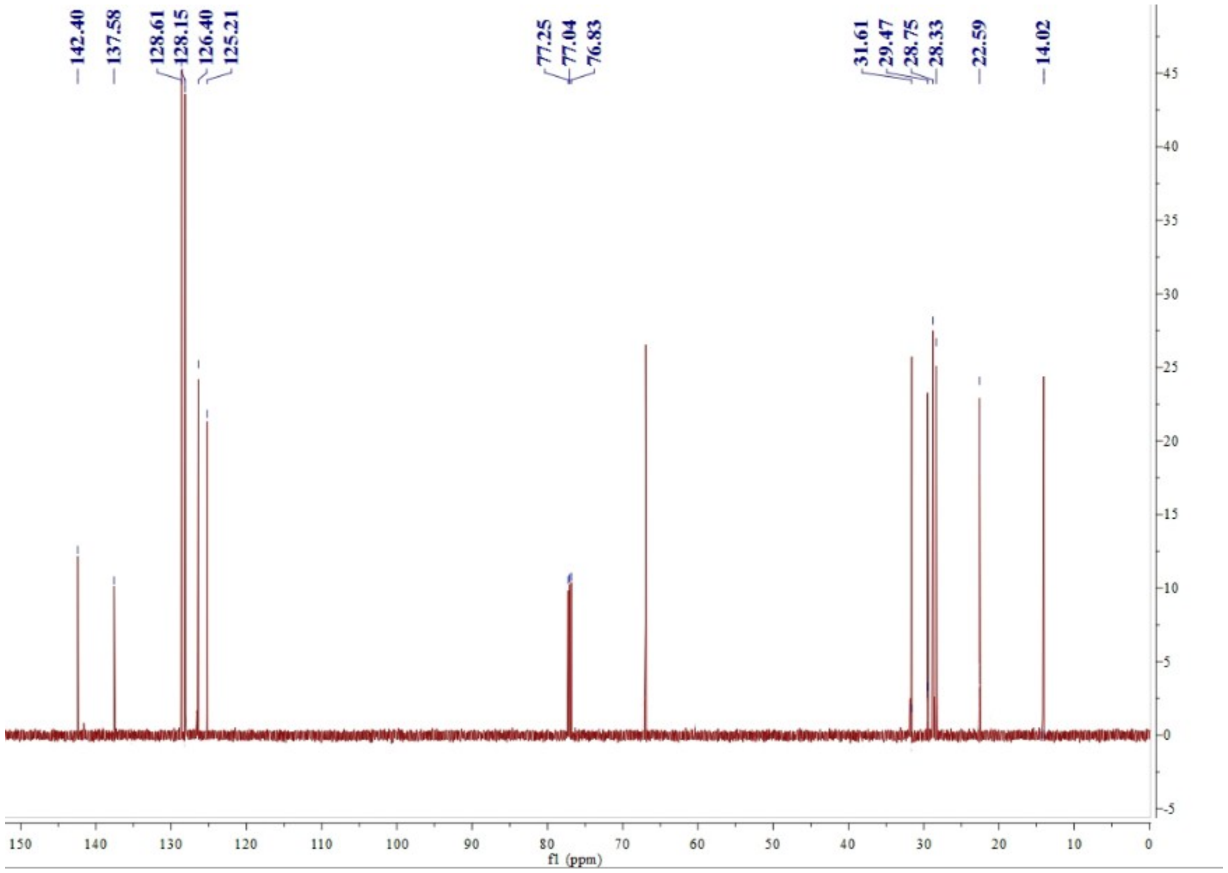

^{1}H NMR of compound 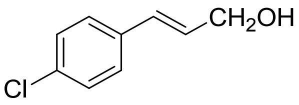

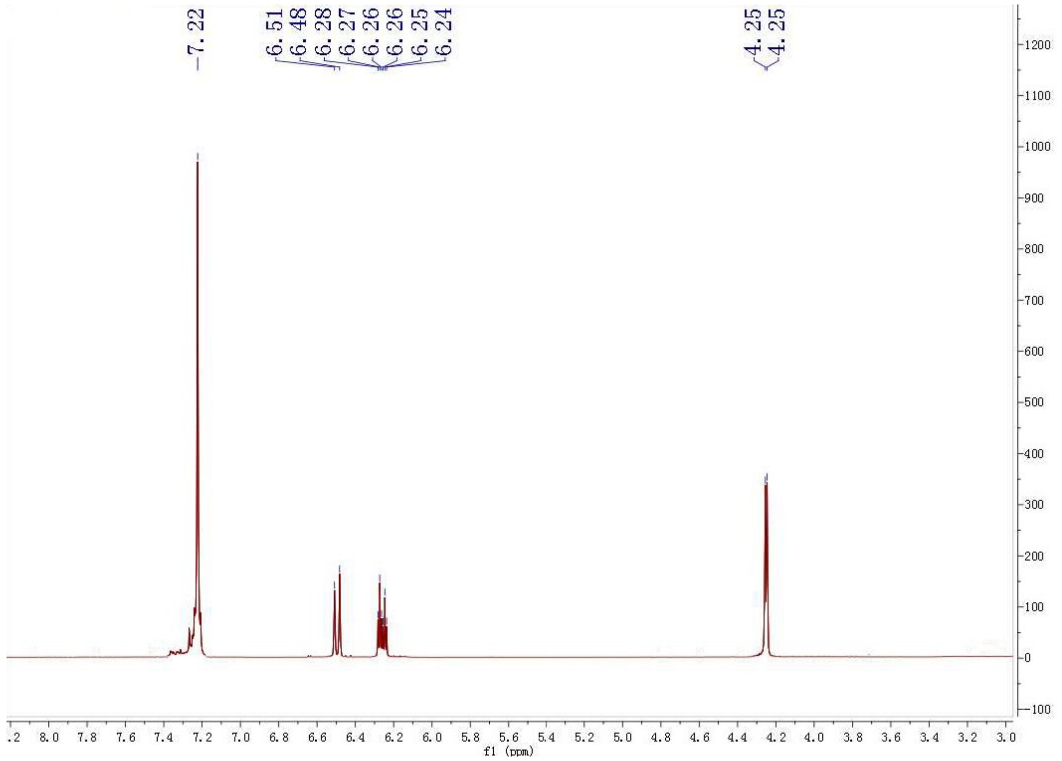

^{13}C NMR of compound 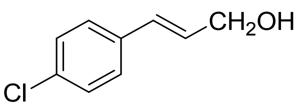

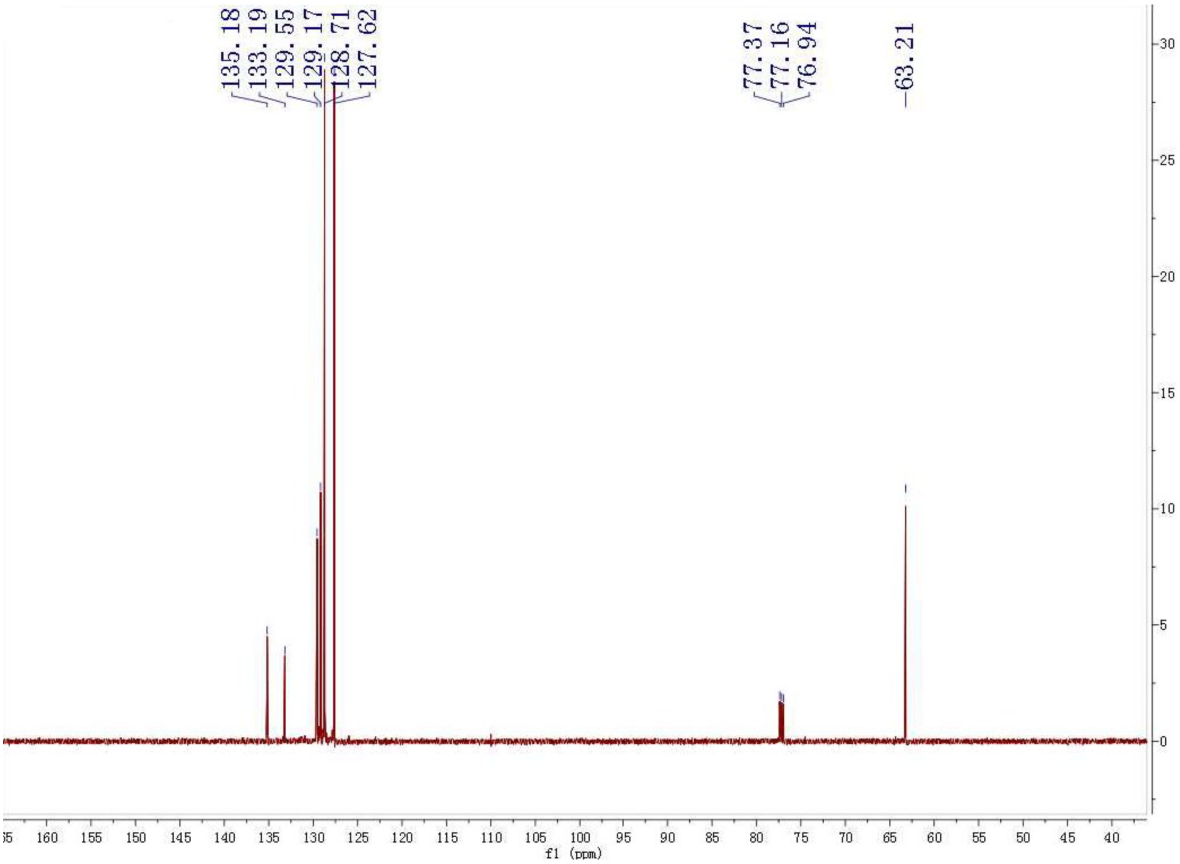

^{1}H NMR of compound 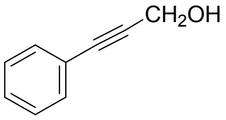

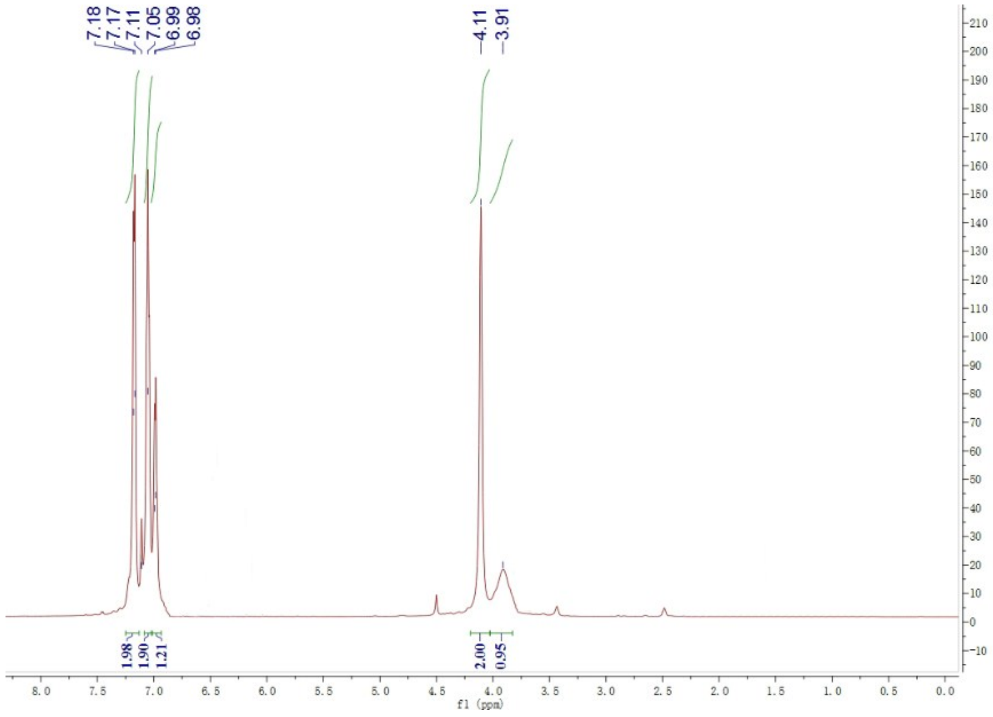

^{13}C NMR of compound 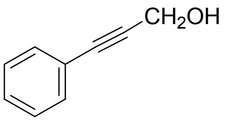

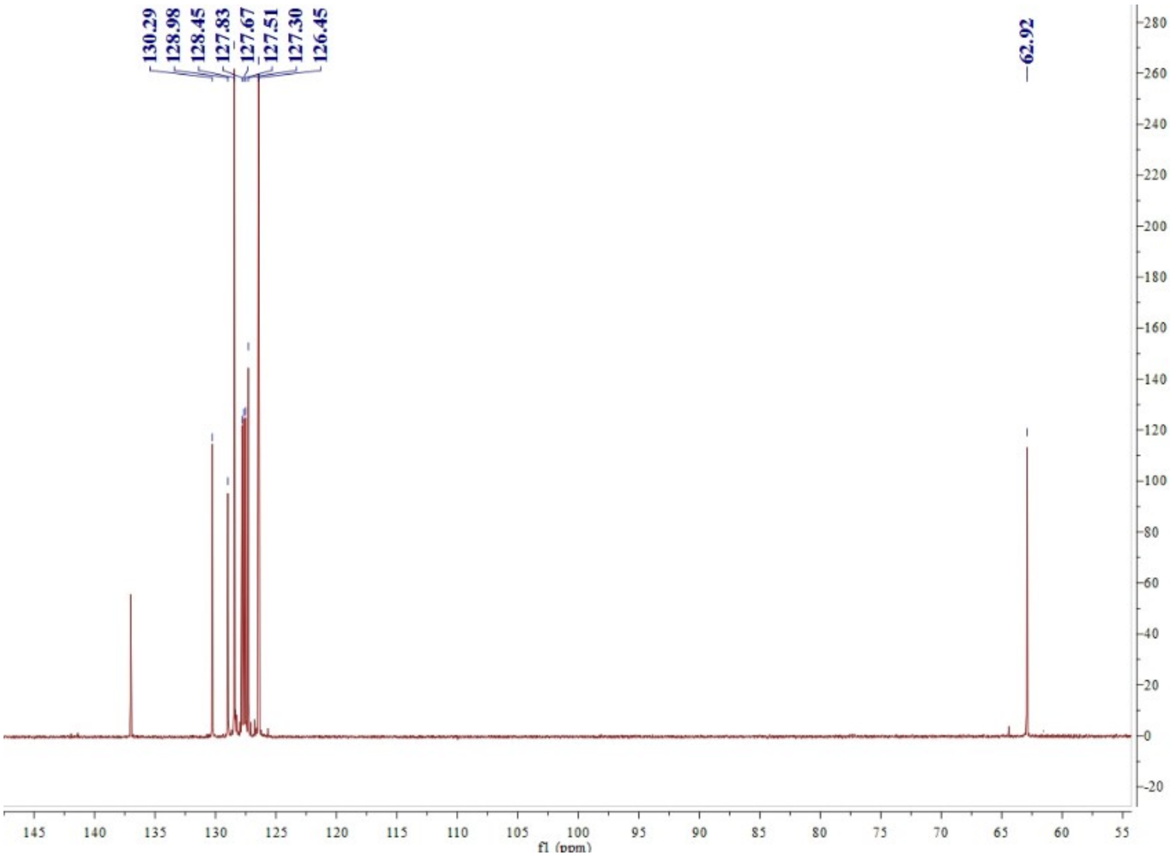

Supplement: RA-008-C8RA02606H-s001 [file RA-008-C8RA02606H-s001.pdf]
